# Optimisation of azide–alkyne click reactions of polyacrylates using online monitoring and flow chemistry†

**DOI:** 10.1039/d4py00984c

**Published:** 2025-01-24

**Authors:** Despina Coursari, Spyridon Efstathiou, Lucas Al-Shok, Mia D. Hall, Ahmed M. Eissa, Evelina Liarou, David M. Haddleton

**Affiliations:** a Department of Chemistry, University of Warwick Gibbet Hill Road Coventry CV4 7AL UK d.m.haddleton@warwick.ac.uk; b School of Life Sciences, Faculty of Science and Engineering, University of Wolverhampton Wolverhampton WV1 1LY UK; c Department of Polymers, Chemical Industries Research Division, National Research Centre Cairo Giza 12622 Egypt

## Abstract

Herein, the online and inline ^1^H-NMR monitoring of azide–alkyne click reactions of polymers is investigated. The effect of the reaction solvent, ligand and temperature were investigated on small “model” molecules. Azidopropanol and 3-azidopropyl-2-bromoisobutyrate were used as the azide-containing molecules, whereas propargyl alcohol was chosen as the alkyne reactant. The optimal conditions were found to be dimethylsulfoxide (DMSO) as solvent with 2,2′ bipyridine as ligand, which were subsequently employed in the modification of different azide-functionalised polyacrylates with propargyl alcohol. From this, poly(methyl acrylate) was chosen to investigate the more complex conjugation of an alkyne monosaccharide. Finally, the “click” reaction was performed in flow leading to a successful modification in less than 1 h.

## Abstract 2

Herein, the online and inline ^1^H-NMR monitoring of azide–alkyne click reactions of polymers is investigated.

## Graphical abstract

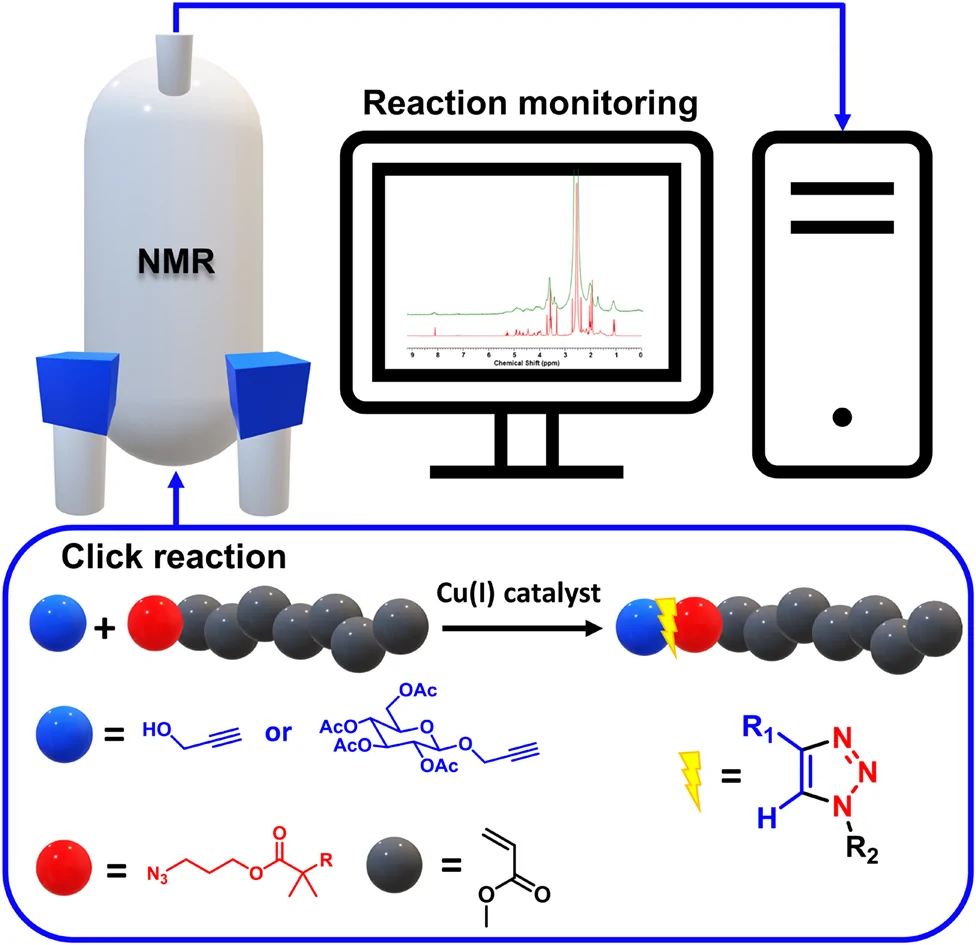

## 1. Introduction

Polymers with chemical diversity are synthesised either by polymerising functionalised monomers or by post-polymerisation modification of reactive polymers. While the first approach has been more common, the second method is gaining popularity for a variety of applications.^1^ Post-polymerisation modification involves the direct polymerisation or copolymerisation of monomers containing chemo selective groups that remain inert under polymerisation conditions but can be efficiently converted in a later step into a variety of other functional groups.^2^ The success of this method relies on its ability to achieve high conversions under mild conditions, its excellent tolerance for different functional groups, and the orthogonality of the post-polymerisation modification reactions. The functional polymers obtained by this approach, maintain consistent chain length and distribution, enabling systematic optimisation for the efficient delivery of genes, small-molecule drugs, protein therapeutics and a variety of applications.^3^

A popular way of post-functionalising is *via* click chemistry. The term “click” chemistry was a term coined by K. Barry Sharpless in 2001 when referring to a family of rapid reactions which are highly selective and reliable without producing any side products.^4^ Clickable polymers can be achieved by using functional initiators or monomers with clickable moieties in controlled radical polymerisations, enabling the construction of various architectures through post-polymerisation clicking. However, the clickable functionality must not interfere with the polymerisation or needs to be protected to maintain control over the process, ensuring well-defined polymers with high functional group fidelity. Many functional monomers or initiators are not commercially available and must be synthesised beforehand. This approach offers flexibility in constructing different functional polymers from a single batch of clickable macromolecules, while maintaining chain length and molar mass distribution for better comparison of functional changes. Post-polymerisation click provides higher functional group fidelity than post-modification, as each repeating unit in the polymer bears the clickable moiety, unlike pre-functionalised homopolymers that require post-polymerisation modification. Additionally, using functionalized initiators ensures mono-terminal functionalisation, whereas post-modification is limited by the yield of the final step.^5–13,15^

An increasing interest has been developed in recent years on carrying out this reaction in flow rather than in batch.^14,15^ Reported advantages are a more efficient heat transfer, higher surface to volume ratio, lower catalyst loadings, more sustainable processes, higher yields and reproducibility.^16,17^ Additionally, avoiding monomer precipitation, large dilution factors, substrate oxidation and offering the opportunity for the progress of the reactions to be monitored in real time.^14,18,19^

To the best of our knowledge, the first CuAAC reaction in flow was reported in 2006 by Wang *et al.* using a microfluidic device for the screening of 32 different click reactions.^20^ Subsequently, further studies followed but very few on the reaction between polymers and carbohydrates with only four found since 2011.^15,21–23^ However, the extent of the reaction was not monitored in any of those cases. A recent click reaction to a carbohydrate in flow was reported by Heida and coworkers in 2020 by functionalising hyaluronic acid (HA) gels.^15^ A microfluidic flow device was utilised at a 25 and 500 μL h^−1^ flow rate with three different click chemistry reactions between HA and poly(ethylene glycol) (PEG) crosslinkers. They investigated UV-initiated thiol–ene, strain promoted alkyne–azide click and a Diels–Alder [4 + 2] cycloaddition reactions.

Polyacrylates are usually soft polymers and often used in biomedicine, cosmetics, fixatives and packaging with click reactions used frequently to modify polymers.^24^ This present study investigates the optimal conditions to understand the click reactions on polyacrylates in flow using nuclear magnetic resonance (NMR)-monitoring of model molecule reactions. Specifically, the reaction of two small azide molecules and propargyl alcohol was followed online by a 400 MHz NMR and a benchtop 80 MHz NMR. Finally, the optimal conditions found were applied when using a benchtop 80 MHz NMR coupled with a flow reactor for inline monitoring of the reaction between azide-containing poly(methyl acrylate) and alkyne derivatised glucose.

## 2. Results and discussion

### 2.1 Solvent effect on the reaction

**Fig. 1:**
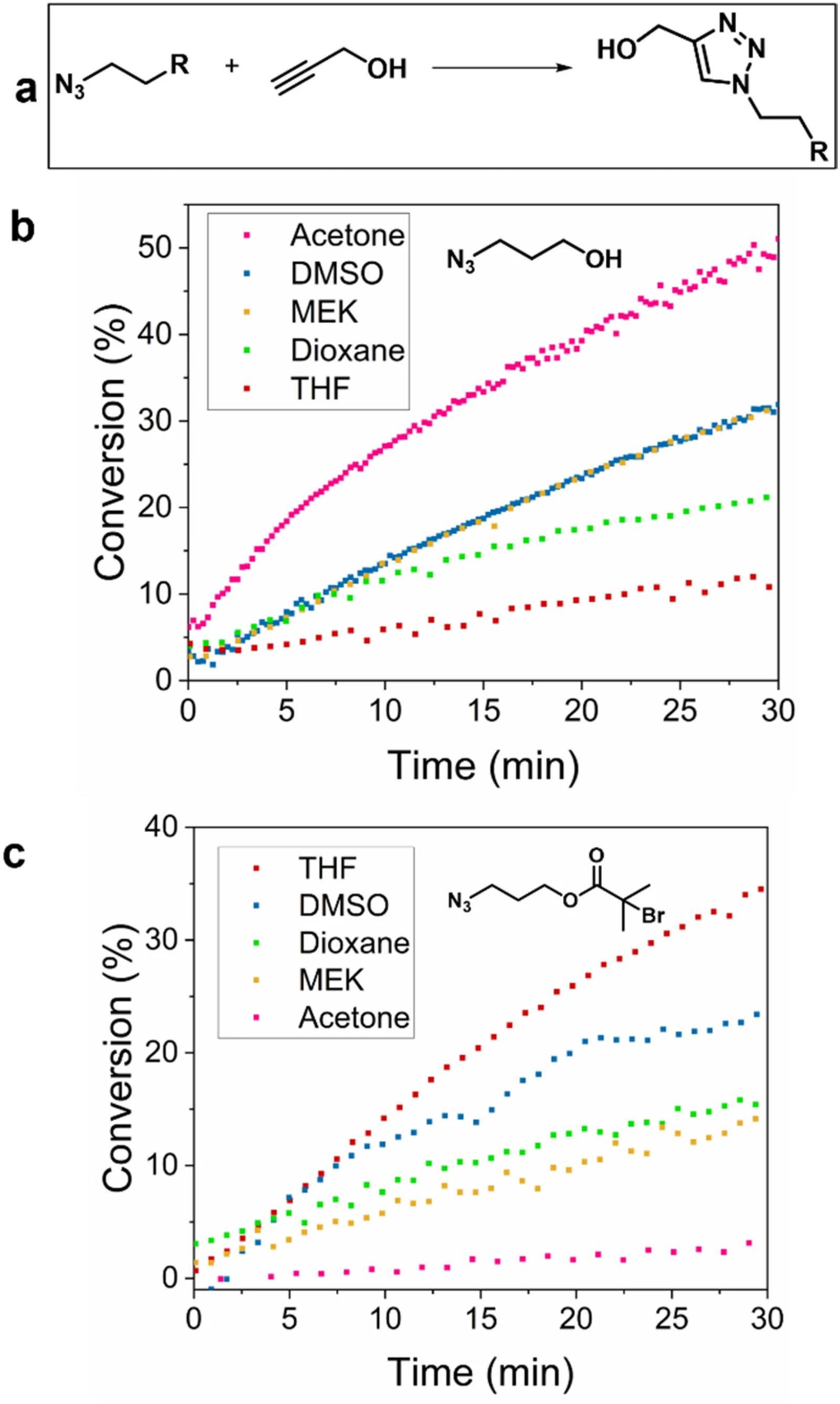
(a) Reaction scheme of the studied “click” reaction. Conversions of the CuAAC reaction with propargyl alcohol in different solvents at 20 °C on a benchtop NMR at 80 MHz (b) with azidopropanol and (c) APBIB.

Aiming to investigate the evolution and parameters of CuAAC, online monitoring of the click reaction between azides and propargyl alcohol was performed in five different solvents, since the choice of solvent plays a significant role in the rate of reaction.^25–27^ Azidopropanol, 3-azidopropyl-2-bromoisobutyrate (APBIB), which are the precursors of the polymers used later, and propargyl alcohol (PrOH), were reacted in different solvents to find the optimum solvent for NMR monitoring, using Cu(i)Br/2,2′-bipyridine (bpy) as the catalyst. Reactions were carried out in a Young's tap NMR tube under nitrogen to minimise oxidation of Cu(i) and monitored using two different NMR instruments, an 80 MHz benchtop (in *protonated* solvents) and a 400 MHz high field instrument. Tetrahydrofuran (THF), acetone, dimethyl sulfoxide (DMSO), dioxane, methyl ethyl ketone (MEK) and chloroform (CHCl_3_) were evaluated as solvents (Fig. 1). The CHCl_3_ peaks overlapped with those of the product therefore, this system was not further investigated. The solubility of the catalyst in each solvent varied as seen by visual observation with the lowest being in dioxane and the highest in DMSO (Fig. S1†). Representative example of the reaction monitoring *via*^1^H-NMR is presented in Fig. S2.† The reaction conversion for azidopropanol and APBIB was determined in five selected solvents and calculated by integrating the peak of the formed triazole (8.0 ppm) against the alkyne proton of PrOH (2–3 ppm depending on the reaction solvent). In DMSO and dioxane, the reaction rate did not change when the azide was altered. For azidopropanol the reaction rate was fastest to slowest for acetone > DMSO, MEK > dioxane > THF while for APBIB the trend was the complete opposite with THF > DMSO > dioxane > MEK > acetone. The observed differences in reactivity trends between the two azides were attributed to their different hydrophobicity with APBIB being more hydrophobic than azidopropanol due to the bromoisobutyrate moiety that decreases polarity. In this case, all chosen solvents were aprotic hence their polarity was speculated to play a role in the reaction rate. However, it is important to note that the reactions monitored in a static NMR tube show different results to a fully stirred reaction as reported by Foley *et al.* In the study they compared the results of heterogeneous and homogeneous reactions monitored both with continuous flow online NMR and static NMR tubes, observing a higher reaction rate in the former.^28^ The study concluded that continuous flow NMR methods give more accurate results for kinetic studies and that in a static NMR tube the reaction is under diffusion control and does not reveal the true kinetic data of the reactions.

### 2.2 Solvent effect on the reaction

**Fig. 2:**
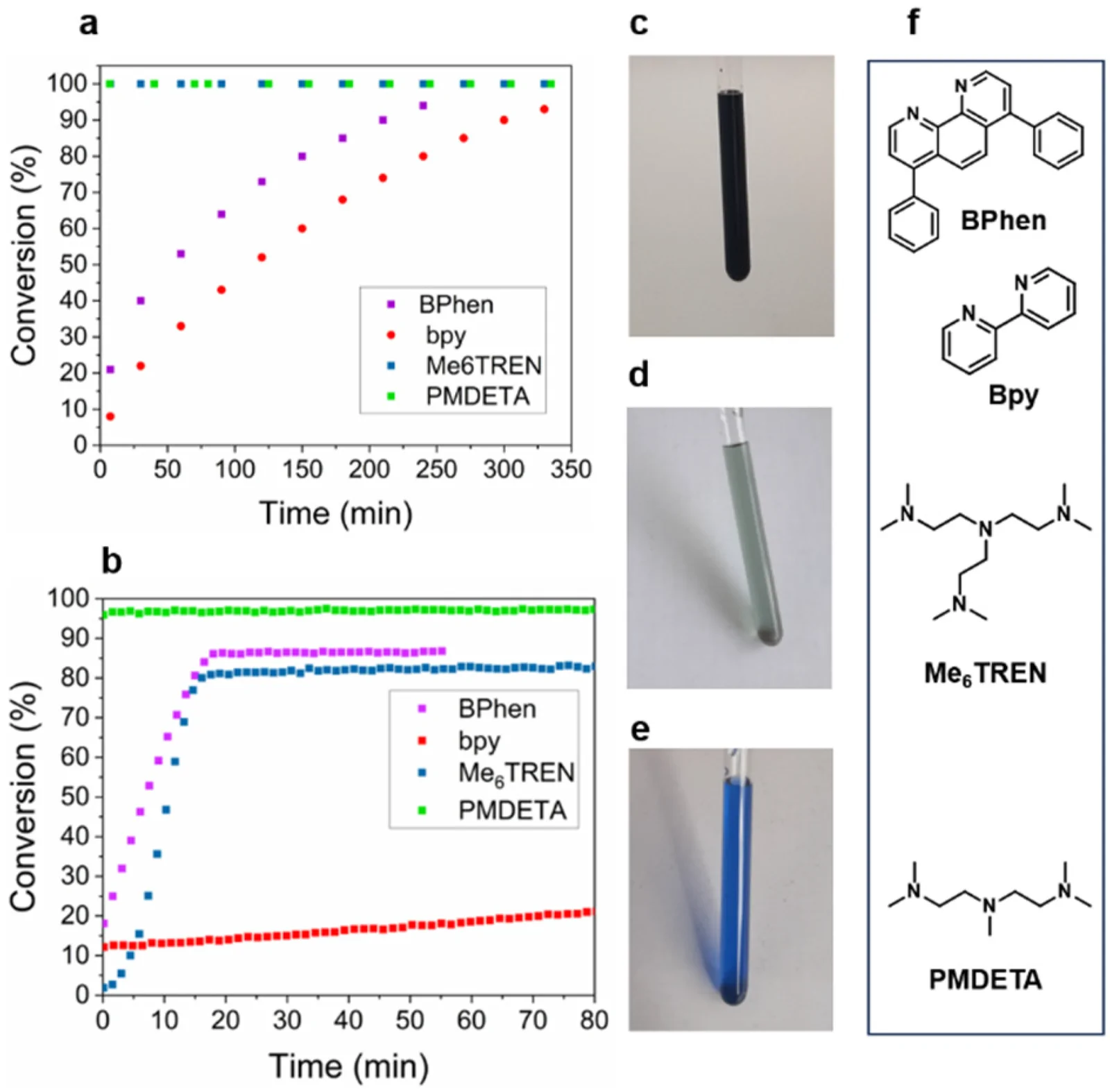
(a) Conversion *vs*. time plot for the “click” reaction with BPhen and bpy ligands at 50 °C on a 400 MHz NMR and (b) conversion *vs.* time of the reaction at 20 °C on a 80 MHz instrument. Catalyst colours in DMSO-d_6_, of (c) bpy and BPhen brown, (d) Me_6_TREN light green and (e) PMDETA blue. (f) Chemical structures of the ligands starting from top; BPhen, bpy, Me_6_TREN, PMDETA.

The solvent of choice was DMSO, as all components were found to be most soluble enabling further reaction monitoring. A further important reaction component is the ligand which alters both the relative stability of Cu(i) and Cu(ii) as well as the solubility. Therefore, reaction rates of azidopropanol with propargyl alcohol were investigated using four structurally different ligands with Cu(i)Br, bpy, bathophenathroline disulfonic acid disodium salt hydrate (BPhen), tris-(2-(dimethylamino)ethyl)amine (Me_6_TREN) and *N*,*N*,*N*′,*N*′′,*N*′′-pentamethyldiethylenetriamine (PMDETA).^29,30^ The reactions were monitored in deuterated DMSO-*d*_6_ at 50 °C using a 400 MHz NMR and at ambient temperature using a 80 MHz benchtop NMR. A monitoring temperature of 50 °C at 400 MHz was chosen following a study by Geng *et al.*, who performed similar reactions reaching maximum conversion in 2 h.^31^ When bpy and BPhen were used, the reaction reached 95% conversion after 6 and 4 h respectively at 50 °C (Fig. 2). When the solvent was added to the Cu(i)-bpy and BPhen catalyst, a dark brown colour was observed due to metal to ligand charge transfer into the low lying π* orbitals (Fig. 2c). In contrast, in the case of the aliphatic ligands Me_6_TREN and PMDETA, the colour observed was light green and dark blue respectively, indicating formation of Cu(ii).^32^ Reaction with the aliphatic ligands reached completion instantly (too fast to monitor using our techniques) (Fig. 2a) attributing this to the disproportionation of Cu(i) to Cu(0) and Cu(ii).^32,33^ The faster reaction with the aliphatic amine ligands Me_6_TREN and PMDETA is attributed to the enhanced formation of a copper–alkyne complex due to enhanced electron-donating properties of the ligand and the corresponding coordination geometry of the Cu complex.^34^ A further reason could be that they are stronger bases (p*K*_a_ Me_6_TREN = 8.99, PMDETA = 9.10)^35^ than the pyridine-based ligands (p*K*_a_ BPhen = 4.27, bpy = 4.33),^36,37^ both reasons based on the proposed mechanism for the Cu(i) catalysis of the cycloaddition.^38–43^ The progress of the reaction was monitored in the presence of Cu(ii)Br_2_ and bpy using the same conditions as with Cu(i) at a ratio of [N_3_] : [Cu(ii)Br] : [bpy] = [1] : [0.2] : [0.4]. The reaction did not proceed, suggesting the requirement for disproportionation to occur (Fig. S4†).^44^ The only change in the NMR spectrum over time was the appearance of broad peaks at *δ* = 5.03 and 4.55 ppm which were previously found to correspond to the hydroxyl protons. This could be an indication that the molecules formed a complex with copper in the beginning of the reaction that starts to break down releasing the molecules in solution, also confirmed by the increase in the intensity of the OH– peak in the NMR spectrum over time.

These reactions were also monitored at 20 °C using a benchtop NMR (80 MHz). The fastest reaction was found to be with PMDETA as ligand, whereby the time the first NMR spectrum was acquired, (approximately 3 min after mixing), the reaction had already reached completion. The second fastest reaction occurred when Me_6_TREN was used as the ligand reaching full conversion at ∼15 min. When BPhen was used as the ligand, the reaction was unexpectedly faster than when monitored at 50 °C reaching completion after 17 min in contrast to 4 h at 50 °C on the 400 MHz. Finally, the reaction with bpy was the slowest with conversions reaching 19% after 1 h at 20 °C and 22% after 50 min at 50 °C. While PMDETA and Me_6_TREN demonstrated faster kinetics, this made reaction monitoring by NMR challenging. Thus, for the rest of this study bpy was used as the preferred ligand for its slower kinetics to allow for reaction monitoring. It should be highlighted that bpy was chosen over BPhen for cost-effectiveness.

### 2.3 Effect of polymer type on reaction rate

**Fig. 3:**
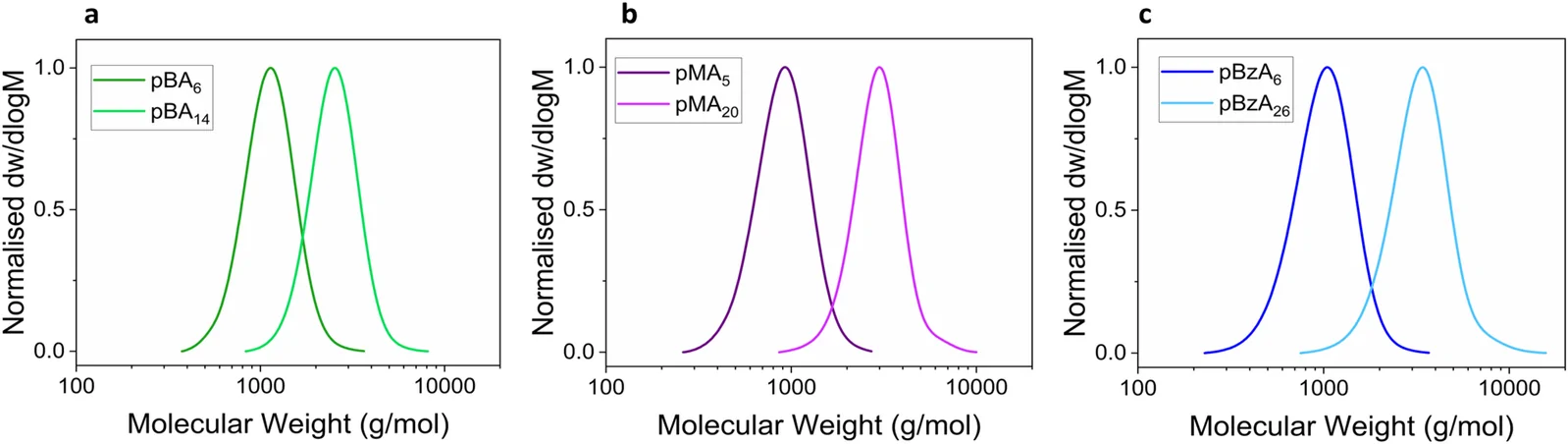
SEC DRI traces of (a) N_3_pBA_6_, N_3_pBA_14_ (b) N_3_pMA_5_, N_3_pMA_20_ and (c) N_3_pBnA_6_ and N_3_pBnA_26_ in CHCl_3_. Molecular weights were calculated according to PMMA standards for a, b and pSt for c.

The optimised conditions, DMSO as solvent and bpy as ligand, were then applied to the click reactions of PrOH with azide-modified small polymers with degrees of polymerisation (DP) < 30, poly(methyl acrylate) (N_3_pMA), poly(butyl acrylate) (N_3_pBA) and poly(benzyl acrylate) (N_3_pBnA) synthesised by Cu(0)-mediated reversible deactivation radical polymerisation (Cu(0)-RDRP) a suitable platform to obtain well-defined polymers with low molecular weights and narrow dispersity.^45,46^ End-group functionalisation was preferred against side-chain as a more predictable and accessible approach for modification. Furthermore, end-group visibility by NMR was another factor in avoiding broad proton peaks which induce errors during integration. The family of acrylates was chosen since polymerisations are faster than *e.g.* methacrylates aligning with the focus to study the post-modification and not the polymerisation process. The three polymers were specifically selected to obtain a range of polymers with varying glass transition temperatures (*T*_g_) exploring this polymer property effect in click reactions. In addition, these polymers lacked reactive moieties in their side chains thus simplifying the system and avoiding interferences with the metal catalyst (*e.g.* possible coordination of the side groups). APBIB was chosen as the initiator in order to obtain polymers with an azide moiety which can be later used to click functional molecules. This azide-functionalised initiator was prepared following a two-step process modified from literature (Scheme S1†).^47^ The conditions used were [I] : [CuBr_2_] : [Me_6_TREN] = [1] : [0.05] : [0.18] as previously reported for the successful homopolymerisation of MA with conversions ranging from 5 to 99.9% depending on the ligand and reaction temperature used.^48,49^ Polymerisations were conducted in DMSO at ambient temperature. The effect of the *T*_g_ on the reaction time was additionally investigated, with N_3_pBA polymers demonstrating the lowest and N_3_pBnA polymers the highest *T*_g_ values as measured *via* dynamic scanning calorimetry (DSC). DSC together with Fourier-transform infrared (FT-IR) spectra are presented in the ESI (Fig. S6 and S7†). Size exclusion chromatography (SEC) graphs of the synthesised polyacrylates are presented in Fig. 3, together with their average molecular weight data (Table 1). The analysis revealed narrow monomodal and symmetrical molecular weight distributions, with a clear shift to higher molecular weights for the higher DP.

**Table 1:** Polymerisation data for the synthesised polyacrylates in DMSO at ambient temperature

Polymer	Reaction time	Conv. (%)	*M*_n th_ a (g mol^−1^)	*M*_n NMR_ b (g mol^−1^)	*M*_n SEC_ c (g mol^−1^)	DP_targ._	DP_NMR_	*Đ* c
N_3_pBA_6_	3 h	99	890	1020	900	5	6	1.14
N_3_pBA_14_	3.5 h	99	1520	2040	2300	10	14	1.19
N_3_pMA_5_	45 min	94	650	680	800	5	5	1.12
N_3_pMA_20_	1 h	>99	1950	1970	2800	20	20	1.10
N_3_pBnA_6_	2 h	98	1040	1220	900	5	6	1.15
N_3_pBnA_26_	2 h	98	3430	4470	3100	20	26	1.15

a *M*_n th_ = [(MWt Monomer × DP_targ._) × Conv.] + MWt APBIB.

b *M*_n NMR_ = [(MWt Monomer × DP_NMR_) + MWt APBIB].

c Obtained by SEC analysis using CHCl_3_ as the eluent and compared against narrow PMMA standards for pBA, pMA and pSt standards for pBnA.

**Fig. 4:**
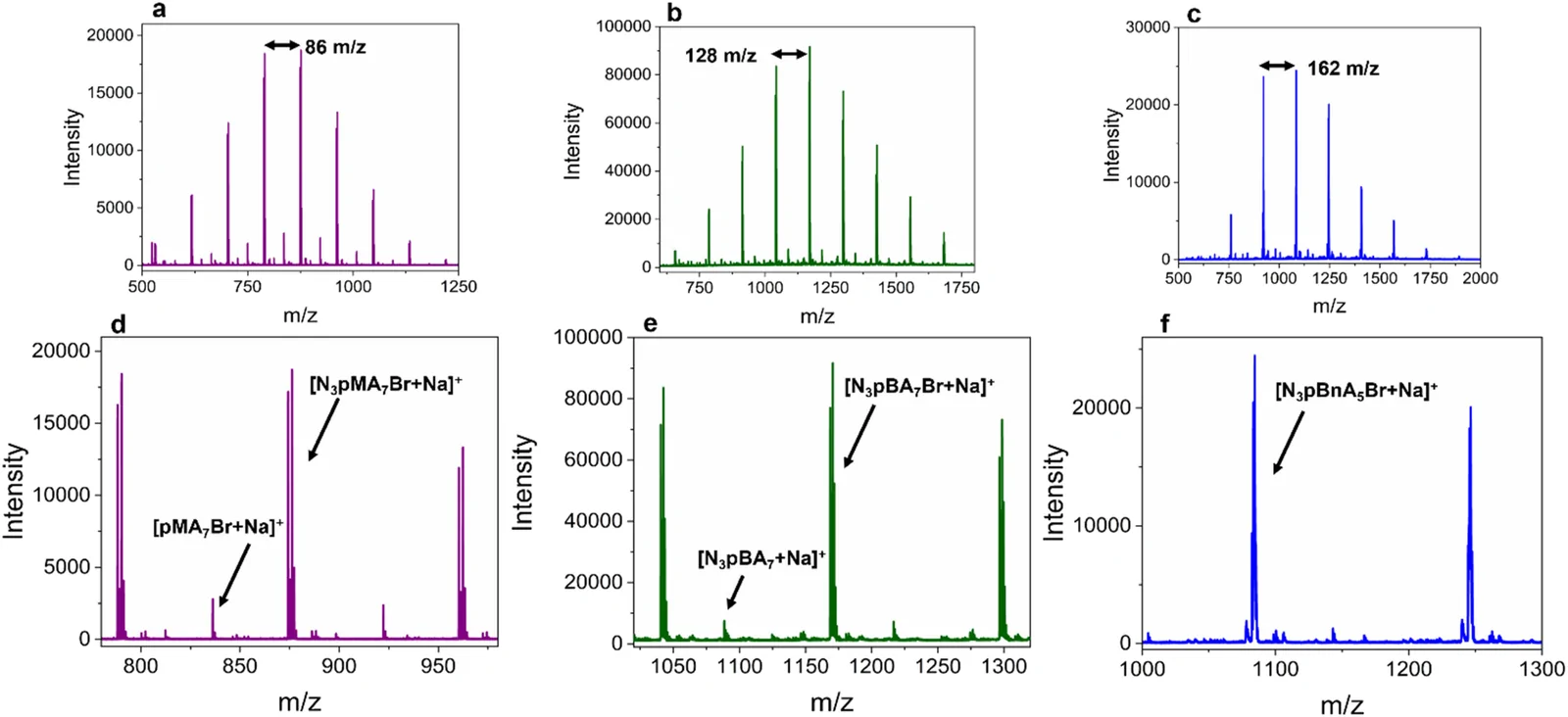
MALDI TOF MS spectra of the three synthesised polyacrylates in THF using *trans*-2-[3-(4-*tert*-butylphenyl)-2-methyl-2-propyldene] malonitrile as a matrix. (a) N_3_pMA_5_, (b) N_3_pBA_6_, (c) N_3_pBnA_6_ (d) zoomed area for N_3_pMA_5_, (e) zoomed area for N_3_pBA_6_, (f) zoomed area for N_3_pBnA_6_.

The polymers were further characterised by matrix assisted laser desorption ionization time of flight mass spectrometry (MALDI-TOF MS), Fig. 4. In all the polymers, the highest intensity peak series corresponded to the azide-bromine terminated polymer, with each peak of the series having a distance of one repeat unit, with the next. Lower intensity peaks were assigned to the polymers with bromine and no azide moiety and *vice versa*. The partial or the full cleavage of the azide group, with loss of molecular nitrogen, are events that have been previously observed and are attributed to the characterisation technique (laser wavelength, matrix)^50^ and not the polymerisation.^51,52^

For the post-polymerisation modification of the synthesised polymers with azide functionality, polymer solutions in DMSO were prepared (0.06 M) with a catalyst ratio of [N_3_] : [alkyne] : [CuBr] : [bpy] = [1] : [1.2] : [0.6] : [1.2]. NMR spectra were acquired every 5 min for a total of 5 h presented in the ESI for each polymer (Fig. S8–S13†). By comparing the observed conversions, the fastest reaction was with N_3_pBnA_6_ and the slowest with N_3_pBA_6_. Therefore, we can conclude that both the chain length and type of homopolymer affect the reaction rate at a certain extent, with no direct correlation to the nature of the polymers. Only in the case of pBnA_6_ a clear difference was observed with a low reaction time.

### 2.4 “Click” of alkyne glucose in flow

**Scheme 1:**
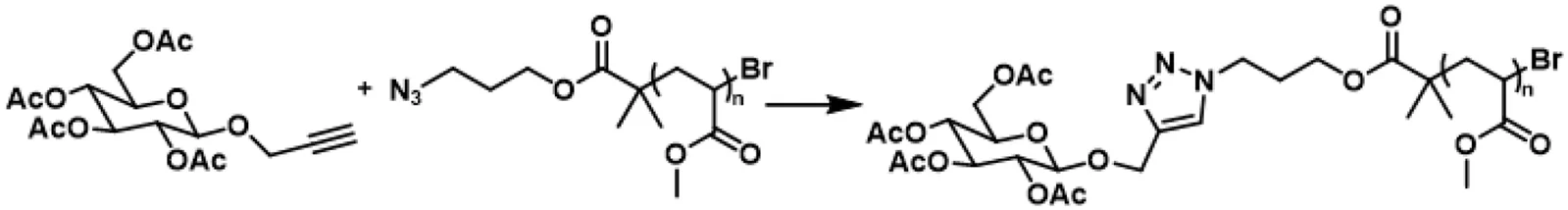
Click reaction of the alkyne functionalised glucose on N_3_pMA.

Following the goals of this study, the last step was the investigation of these reactions in flow. The optimal conditions found were applied when using a benchtop 80 MHz NMR coupled with a flow reactor for inline monitoring of the reaction between N_3_pMA and an alkyne functionalised glucose (Scheme 1). Glucose was chosen due to its biological significance and well-studied chemical properties making it an ideal model molecule for functionalisation.

Furthermore, it was selected for a potential application in lectin-binding studies for treatment or diagnosis. It should be also highlighted that the choice to use low MW polymers for inline monitoring of the click reaction was intentional. High MW polymers would potentially result to weak end-group proton peaks complicating NMR monitoring while the higher viscosity would probably result into flow issues requiring different strategies to achieve high conversion rates.

**Fig. 5:**
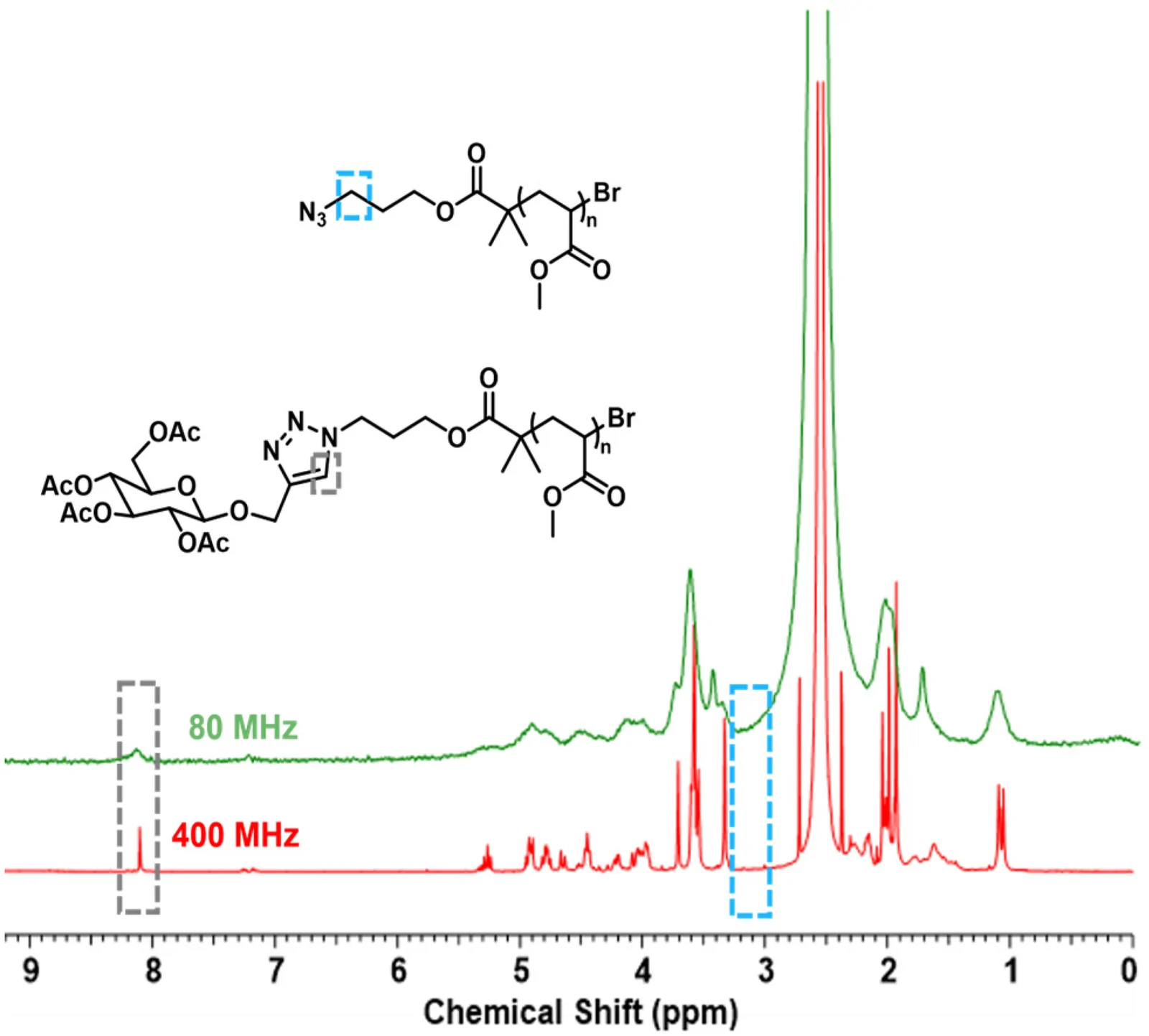
^1^H-NMR spectra in DMSO of N_3_pMA_5_ (0.18 M) using an 80 and a 400 MHz instrument after 42 min of the reaction with GlcAcPr in flow.

Initially, modified propargyl glucose was *clicked* on the N_3_pMA_5_ homopolymer in an NMR tube to assess if the concentration of reactants was adequate for monitoring, with the reaction reaching 41% conversion after 1.5 h at a polymer concentration of 0.18 M and an equivalence of [N_3_pMA_5_] : [CuBr] : [bpy] = [1] : [0.2] : [0.4]. Finally, the same conditions (DMSO, bpy) were used to conduct the reaction in flow using a Vapourtec® E series to assess the occurrence of the reaction under flow (Fig. S14†). Subsequently, N_3_pMA_5_ with a catalyst equivalence [N_3_pMA_5_] : [CuBr] : [bpy] = [1] : [0.2] : [0.4] was reacted with propargyl glucose tetraacetate (GlcAcPr, 1.2 eq.) in a flow reactor. The mixture was first left at ambient temperature for 15 min to mimic the conditions of the reaction in an NMR tube as the Bruker 400 MHz needed 15 min for the reaction mixture to reach 50 °C. The flow stream was set at 0.25 mL min^−1^ to the reactor with polytetrafluoroethylene (PTFE) tubes of 8 mL capacity at 50 °C and a reaction residence time of 32 min. The reaction was subsequently monitored by ^1^H NMR with spectra acquired using 32 scans and a 6.4 s acquisition time at approximately 42 min after starting the flow stream. The NMR spectra obtained from the 80 MHz (inline) and the 400 MHz (offline) are presented in Fig. 5. The absence of the N_3_CH_2_– proton peak at *δ* = 3.30 ppm indicated that the reaction had reached full conversion after 1 h. When the reaction was performed in the NMR tube it only reached 38% conversion after 1 h at 50 °C showing that the reaction in flow is more efficient for the click reaction.

## 3. Conclusions

In the present study the optimal conditions for the NMR-monitoring of the “click” reaction of two small azide molecules and propargyl alcohol were investigated; DMSO was found to be the optimum out of five other solvents owing to the efficient solubility of the reaction components and reaction rates suitable for monitoring. BPhen and bpy showcase similar results at 50 °C however BPhen was considered a quite expensive ligand to use. The use of aliphatic amine ligands Me_6_TREN and PMDETA led to rapid reactions with almost instant completion which were not possible to be monitored with neither the inline flow 80 MHz NMR nor online on a 400 MHz NMR instrument. The polymer type effect was also investigated with no correlation between chain length and monomer type on the reaction speed. From the six polymers compared, the one that gave the highest reaction rate was pBnA_6_. NMR monitoring using a 80 MHz inline system has proven to be beneficial for fast reaction screening and optimisation, with possibility to couple with a flow reactor, with sufficient resolution for reaction monitoring if the correct concentration of reactants is used.^53^

The contextualisation of the findings of this study against prior literature confirms some known patterns but also highlights important differences. For example, a study by Golas *et al.* on optimising the step-growth click coupling of low MW α,ω-diazido-terminated polystyrene reported an 85% conversion using PMDETA and Me_6_TREN in DMF at 25 °C after 30 min resulting in slower conversion rates compared to our findings (3 min full conversion for PMDETA and 15 min full conversion for Me_6_TREN).^54^ Reaction with bpy led to a 5% conversion in 30 min in contrast to 15% for the same time in our study. Both studies confirmed the slower reactivity of bpy compared to aliphatic ligands. In another study by Geng *et al.*, CuAAC reactions between alkyne-functionalised glycopolymers and sugars were performed in DMSO at 50 °C using BPhen.^55^ The cycloadditions reached full conversion within 2 h in contrast to our systems that required 4.5 h to achieve full conversion suggesting potential differences in reagent purity, catalyst preparation or experimental setup. DMSO was also chosen in these reactions due to its excellent solubility assuring all components remain in solution. Finally, a comparison with a study from Bell *et al.*, towards the synthesis of highly branched polymers using CuAAC showed faster reaction conditions with PMDETA (85% in 10 min) compared to Me_6_TREN (55% in 10 min) in a mixture of DMSO and toluene at 25 °C.^56^ Same trend were also observed in our study at 20 °C reinforcing that PMDETA outperforms Me_6_TREN under comparable conditions.

## Author contributions

DC carried out the experiments, conceptualised experiments, performed data analysis and wrote this manuscript. SE and AME helped with execution and conceptualisation. SE also assisted in manuscript corrections and reviewing. LAS performed the MALDI-TOF analysis and helped with conceptualisation. MDH performed the flow reaction. EL assisted on the conceptualisation and writing of manuscript. DMH conceived the original idea, acquired funding, helped write the final version of the manuscript and supervised the project.

## Conflicts of interest

DC There are no conflicts to declare.

## Supplementary Material

PY-016-D4PY00984C-s001

## Data Availability

The data supporting the findings of this study are available within the article and its ESI.† Any additional datasets generated and/or analyzed during the current study are available from the corresponding author upon reasonable request.
