# Supplementary material for: Optimisation of azide–alkyne click reactions of polyacrylates using online monitoring and flow chemistry
Source: Polym Chem. 2025 Jan 24;16(9):1065–71. doi: 10.1039/d4py00984c (PMC13580200; doi:10.1039/d4py00984c)
Supplement: Supplementary file 1 [file PY-016-D4PY00984C-s001.pdf]

Supplementary Information (SI) for Polymer Chemistry.
This journal is © The Royal Society of Chemistry 2025

Electronic Supplementary Information

Optimisation of azide-alkyne click reactions of polyacrylates using online monitoring and flow chemistry

Despina Coursari[a], Spyridon Efstathiou [a], Lucas Al-Shok[a], Mia D. Hall[a], Ahmed M. Eissa[a,b,c], Evelina Liarou [a], David M. Haddleton [*a]

*Corresponding author

[a] Department of Chemistry, University of Warwick Gibbet Hill Road, CV4 7AL Coventry, UK.

[b] School of Life Sciences, Faculty of Science and Engineering, University of Wolverhampton, Wolverhampton WV1 1LY, UK.

[c] Department of Polymers, Chemical Industries Research Division, National Research Centre, Cairo Giza 12622, Egypt.

Table of Contents

1 Experimental Section 2
1.1.1 Materials 2
1.1.2 Instrumentation 2
1.1.3 Synthesis 3
2 Supplementary analysis and experimental data 7
2.1 Solvent effect on the reaction 7
2.2 Effect of the ligand on the reaction rate 8
2.3 Effect of the polymer type on the rate of reaction 9
2.5 Results from in batch ^{1}H-NMR monitoring 10
2.6 Flow reaction set-up 13
3 References 14

1 Experimental Section

1.1.1 Materials

2,2'-Bipyridine (bpy ≥ 99%, Acros Organics), bathophenathrolinedisulfonic acid disodium salt hydrate (bathophen.), *N,N,N',N'',N''*-Pentamethyldiethylenetriamine (PMDETA ≥ 99%), trhiethylamine (TEA 99%), dichloromethane (DCM ≥ 99%,), dimethyl sulfoxide (DMSO ≥

99%, and DMSO-d_6), dioxane, acetone, methyl ethylketone (MEK), tetrahydrofuran (THF 99.5% GC grade), propargyl alcohol (PrOH), β-D-Glucose pentaacetate (98%, Lancaster) were purchased from Sigma Aldrich and used as received unless otherwise stated. Methyl acrylate (MA 99%, Sigma Aldrich), butyl acrylate (BA 99%, Sigma Aldrich), benzyl acrylate (BnA 97%, Alfa Aesar) were used as received, without subsequent purification. Tris-(2-(dimethylamino)ethyl)amine (Me_6Tren) was synthesized according to previously reported literature.[1] Copper(I) bromide (Cu(I)Br, 98%, Sigma-Aldrich) was washed with acetic acid and ethanol and dried under vacuum prior to use.[2] Azidopropanol and APBIB were synthesised according to previously reported literature.[3, 4]

1.1.2 Instrumentation

NMR Spectroscopy. ^{1}H NMR spectra were recorded on a Bruker Avance 400 MHz spectrometer at 20 °C or at 50 °C, with a 90 sec acquisition time and 16 scans per spectrum. From mixing until acquisition time there was a 20 min delay causing an expected lag in the kinetic.

Benchtop NMR. Reactions were monitored *via* ^{1}H NMR on a Spinsolve Magritek 80 MHz instrument. Spectra were obtained every 33 sec for a maximum period of 1 hr with acquisition time of 3.2 sec and 2 scans. For the ligand comparison spectra were taken every 71 sec for a maximum period of 10 hours with an acquisition time of 3.2 sec and 4 scans.

Differential scanning calorimetry. DSC measurements were performed on a Mettler Toledo DSC1-STARe system under nitrogen flow (50 mL min^{-1}). Samples were loaded into 40 μL aluminium pans. Methods varied depending on the sample used and T_g values were calculated from the midpoint of the thermogram from the second heating cycle unless otherwise stated.

FT-IR Spectroscopy. FT-IR spectroscopy measurements were carried out using a Shimadzu IR Spirit spectrometer. Spectra were recorded from 4000 to 400 cm^{-1} with 8 scans performed for each sample.

SEC analysis. $CHCl_3$ was used as the mobile phase on an Agilent Infinity II MDS instrument equipped with a PLgel 5 µm guard column, 2 × PLgel Mixed C columns (300 × 7.5 mm), differential refractive index (DRI), viscometry (VS), dual angle light scatter (LS) and multiple wavelength UV detectors. Samples were run at a flow rate of 1 ml/min at 30 °C. The experimental values were determined by conventional calibration (according to narrow PMMA standards in the range 550 to 1,591,000 g/mol and narrow pSt standards in the range of 160 to 364,000 g/mol) using Agilent GPC/SEC software.

MALDI-ToF MS. Matrix assisted laser desorption ionization time-of-flight mass spectrometry was conducted using a Bruker Daltonics Ultraflex II MALDI-ToF mass spectrometer, equipped with a nitrogen laser delivering 2 ns laser pulses at 337 nm with positive ion ToF detection performed using an accelerating voltage of 15-30 % kV. Solutions in tetrahydrofuran (THF) of trans-2-[3-(4-tert-butylphenyl)-2-methyl-2-propyldene] malonitrile (DCTB) as a matrix (saturated solution), sodium iodide as the cationization agent (12 mg/mL) and sample (0.025 mg/mL) were mixed and then spotted to the target plate. Spectra were recorded in linear positive mode calibrated with PEG-monomethyl ether 1,900 kDa.

Flow with in-line NMR. The Vapourtec® E series Integrated Flow Chemistry System (R2+) was the platform used for the flow experiment. Flow stream was directed with 0.25 mL/min to

the reactor with PTFE tubes of 8 mL capacity at 50 °C and reaction residence time of 32 min. The reactor was connected to a Spinsolve Magritek 80 MHz instrument and the reaction was monitored with ^{1}H NMR acquired with 32 scans, a 90° pulse angle, and a 6.4 sec acquisition time.

1.1.3 Synthesis

3-Azido-1-propanol.

In the preparation of azides care should be taken to keep the aqueous reaction or workup solutions non-acidic to avoid the generation of volatile, toxic, and explosive HN_3. Unreacted NaN_3 in water was neutralised by oxidation with Ammonium Cerium(IV) Nitrate. Procedure was modified from literature.[3] Sodium azide (6.24 g, 96.0 mmol) and deionised water (60 mL) were added into a 250 mL three-neck round-bottom flask equipped with a condenser. 3-Bromo-1-propanol (4.33 mL, 47.9 mmol) was added dropwise for 10 min, and the reaction mixture was stirred at 70 ˚C for 24 h. The mixture was then extracted with DCM (5 x 60 mL) and the organic layer was dried over anhydrous $MgSO_4$ for 1h. A light-yellow liquid was obtained by the removal of DCM on the rotary evaporator (yield: 4.49 g, 93 %). ^{1}H NMR (400 MHz, $CDCl_3$), δ (ppm): 3.74 (q, 2H, J = 5.8, C**H**$_2$OH), 3.44 (t, 2H, J = 6.6, C**H**$_2$N$_3$), 1.91 (t, 1H, J = 5.0, CH$_2$O**H**), 1.83 (m, 2H, C**H**$_2$CH$_2$OH).

3-Azidopropyl 2-bromoisobutyrate (APBIB).

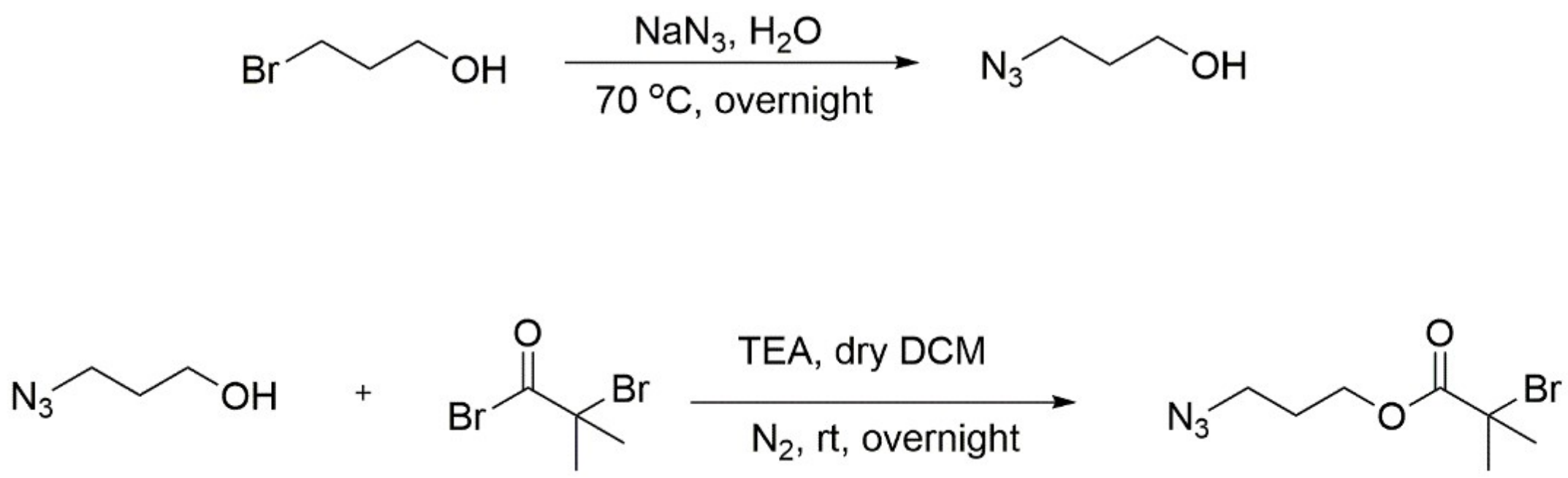

Scheme S1. *Synthesis of the azide-functionalised initiator (APBIB) in 2 steps.*

Procedure was modified from the literature.[4] 3-Azidopropanol (4.49 g, 43.6 mmol), TEA (7.35 mL, 52.7 mmol) and dry DCM (30mL) were added into 250 mL two-neck round-bottom flask equipped with a dropping funnel and the mixture was degassed for 20 min. 2-Bromoisobutyryl bromide (BiB) (5.86 mL, 47.4 mmol) and dry DCM (15 mL) were added into the dropping funnel. The two-neck flask was immersed into an ice-water bath and the mixture was stirred with a magnetic bar under nitrogen atmosphere. Then, over a period of 1 h the BiB solution was added dropwise into the flask. After the addition was completed, the reaction mixture was stirred further at 0 °C for 2 h and at room temperature for another 17 h. The undissolved solid formed was then filtered and the remaining solution was extracted with a saturated solution of $NaHCO_3$ (3 x 50 mL). The organic phase was then dried over anhydrous $MgSO_4$ for 1 h. DCM was removed by rotary evaporation, and the residue was further purified by column

chromatography using petroleum ether:ethyl acetate (95:5 v/v) as eluent. The solvents were removed by rotary evaporation and APBIB was obtained as a light-yellow oil (yield: 6.15 g, 56 %). ^{1}H NMR (400 MHz, $CDCl_3$) δ_{ppm}: 4.28 (t, 2H, J = 6.1, COOC**H**$_2$), 3.45 (t, 2H, J = 6.7, C**H**$_2$N$_3$), 1.97 (m, 2H, C**H**$_2$CH$_2$N$_3$), 1.94 (s, 6H, C(C**H**$_3$)$_2$Br). ^{13}C NMR (400 MHz, $CDCl_3$) δ_{ppm}: 171.42 (C=O), 62.70 (COO**C**H$_2$), 55.69 (CBr), 47.97 (CH_2N_3), 30.65 (CH_3), 27.93 (CH_2). ν_{max}(cm^{-1}): 3287b (OH), 2971 and 2870 (CH_2), 2094 (N_3).

APBIB-pMA with targeted DP = 5.

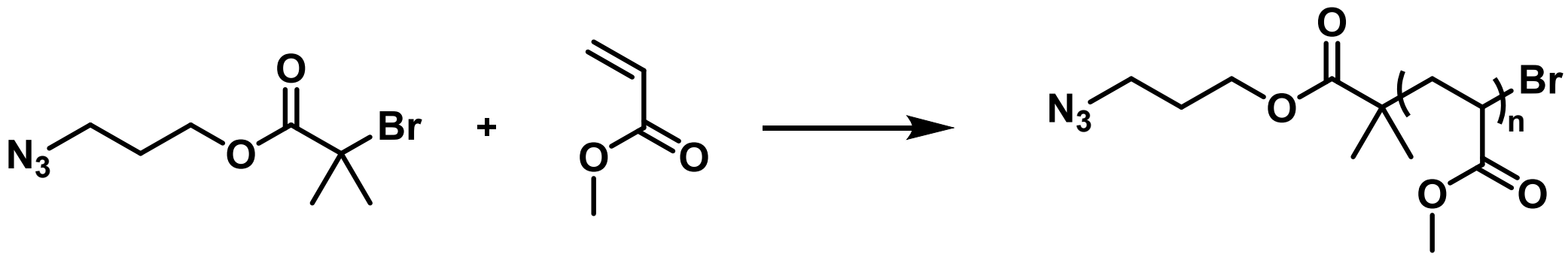

Scheme S2. *Polymerisation of MA.*

MA (1 mL, 5 eq.), APBIB (391 µL, 1 eq.), Cu(II)Br$_2$ (24.8 mg, 0.05 eq.), Me$_6$TREN (107 µL, 0.18 eq), DMSO (2 mL) and copper wire (5 cm) wrapped around a stirring bar were added to a septum sealed vial of 8 mL and the mixture was deoxygenated by bubbling N_2 for 15 min. The polymerisation was allowed to commence at ambient temperature and the conversion was determined by ^{1}H NMR by comparison of the integration peaks from the monomer (vinyl protons, 5.75 ppm) with peaks from the backbone ($COOCH_3$, 3.71 and 3.61 ppm). Same procedure was followed for targeted DP = 20. The polymers were purified by precipitation in water/ methanol (120 mL, 4:1), dissolution in THF and removal of the solvent by rotary evaporation. M_n values were determined from $CHCl_3$ SEC and ^{1}H NMR of the purified polymer by comparison of the integration peaks of the initiator (CH_2N_3, 3.41 ppm) with peaks from the backbone ($COOCH_3$, 3.78 and 3.67 ppm).

APBIB-pBA with targeted DP = 5.

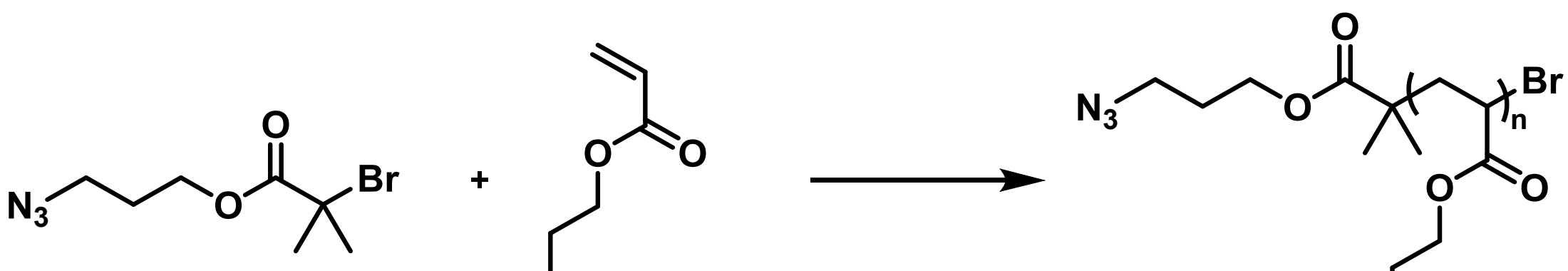

Scheme S3. *Polymerisation of BA.*

BA (1 mL, 5 eq.), APBIB (246 µL, 1 eq.), Cu(II)Br$_2$ (15.6 mg, 0.05 eq.), Me$_6$Tren (67.1 µL, 0.18 eq.), DMSO (2 mL) and copper wire (5 cm) wrapped around a stirring bar were added to a septum sealed vial of 8 mL and the mixture was deoxygenated by bubbling with N_2 for 15 min. The polymerisation was allowed to commence at ambient temperature and the conversion was determined by ^{1}H NMR by comparison of the integration peaks from the monomer (vinyl protons, 5.75 ppm) with peaks from the backbone (CH_2CH_3, 0.87 ppm). Same procedure was

followed for targeted DP = 10. The polymers were purified by precipitation in water/ methanol (120 mL, 4:1), dissolution in THF and removal of the solvent by rotary evaporation. M_n values were determined from $CHCl_3$ SEC and ^1H NMR of the purified polymer by comparison of the integration peaks of the initiator (CH_2N_3, 3.40 ppm) with peaks from the backbone (CH_2CH_3, 0.95 ppm).

APBIB-pBnA with targeted DP = 5.

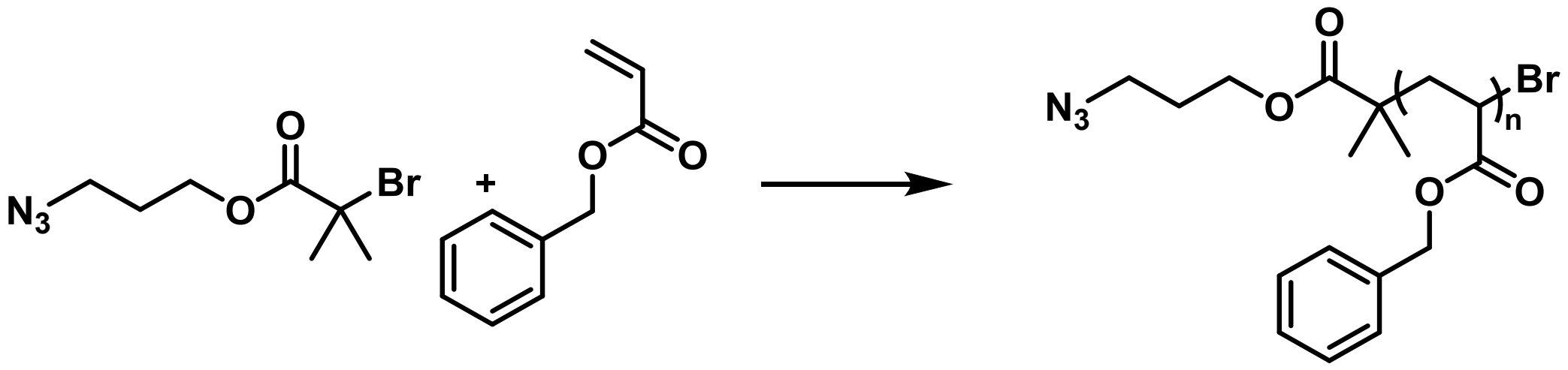

Scheme S4. *Polymerisation of BnA.*

BnA (1 mL, 5 eq.), APBIB (231 µL, 1 eq.), Cu(II)Br_2 (14.7 mg, 0.05 eq.), Me_6Tren (63.2 µL, 0.18 eq.), DMSO (1 mL) and copper wire (5 cm) wrapped around a stirring bar were added to a septum sealed vial of 8 mL and the mixture was deoxygenated with N_2 for 10 min. The polymerisation was allowed to commence at ambient temperature and the conversion was determined by ^1H NMR by comparison of the integration peaks from the monomer (vinyl protons, 5.84 ppm) with peaks from the backbone (C_6H_5, 7.37 ppm). Same procedure was followed for targeted DP = 20. The polymers were purified by precipitation in methanol, dissolution in THF and removal of the solvent by rotary evaporation. M_n values were determined from $CHCl_3$ SEC and ^1H NMR of the purified polymer by comparison of the integration peaks of the initiator (CH_2N_3, 3.41 ppm) with peaks from the backbone (C_6H_5, 7.37 ppm).

Synthesis of alkyne sugars

2-Propynyl-tetra-*O*-acetyl-β-D-glucopyranoside (GlcAcPr).

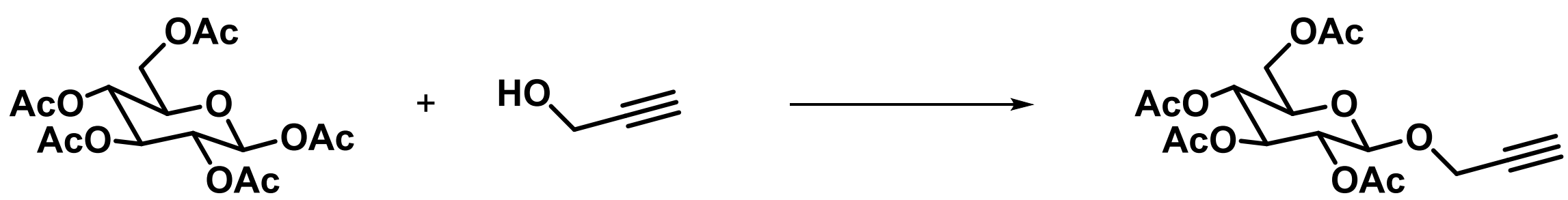

Scheme S5. *Modification reaction of β-D-Glucose pentaacetate.*

Procedure was adapted from the literature.[5] β-D-Glucose pentaacetate (5.0 g, 12.9 mmol) and propargyl alcohol (0.9 mL, 15.5 mmol) were dissolved in dry DCM (40 mL) and the mixture was deoxygenated by bubbling with N_2 for 10 min. The mixture was placed in an ice bath and BF_3-Et_2O (2.4 mL, 19.4 mmol) was added in portions. The reaction was left to stir at ambient temperature for 2 h and was monitored *via* TLC and NMR. Upon completion, anhydrous K_2CO_3 was added, the mixture was stirred further for 30 min and the salt was finally removed by filtration. The filtrate was washed with water (3 x 50 mL), the aqueous phase was separated

and extracted with DCM (2 x 30 mL) and the combined organic phases were dried over anhydrous $MgSO_4$. Volatiles were removed under vacuum and the brown solid obtained was recrystallised twice (DCM : Hexane = 1 : 2) to yield colourless needle-like crystals (yield: 1.60 g, 33 %).^{1}H NMR (400 MHz, $CDCl_3$) δ_{ppm}: 5.25 (m, 1H, **H-4**), 5.10 (m, 1H, **H-3**), 5.02 (m, 1H, **H-2**), 4.78 (d, 1H, J = 7.9, **H-1**), 4.38 (d, 2H, J = 2.3, C**H**$_2$C≡CH), 4.30-4.13 (m, 2H, **H-6**), 3.74 (m, 1H, **H-5**), 2.47 (t, 1H, J = 2.3, CH_2C≡C**H**), 2.09, 2.06, 2.03, 2.01 (4s, 12H, OCOC**H**$_3$). FTIR: ν 3273 (C≡C−H), 2970 (C−H_3) and 1731 cm^{-1} (C=O).

General procedure for the 'click' reaction.

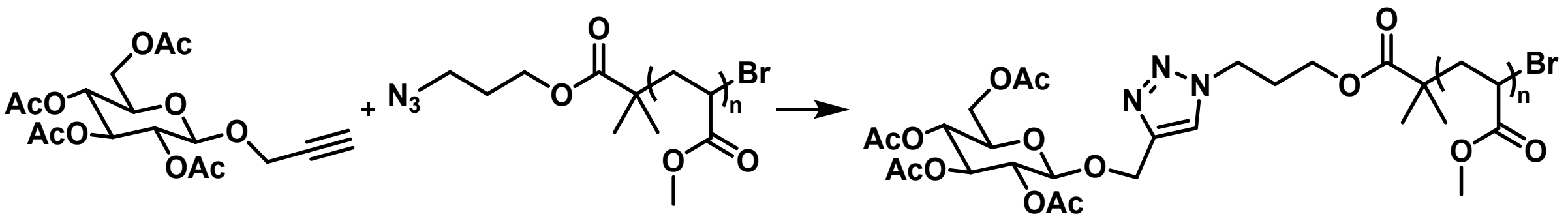

Scheme S6. *"Click" reaction on N_3-pMA.*

Cu(I)Br (3.1 mg, 0.2 eq.) and bpy (6.8 mg, 0.4 eq.) were added in a septum sealed vial and purged with N_2 for 2 min. Then 0.25 mL DMSO-d_6 were added under stirring and the mixture was degassed for 10 min. For liquid ligands, DMSO-d_6 and the ligand were first mixed in a vial and then added to the Cu(I)Br. In a separate vial, azidopropanol (10 µL, 1 eq.) and propargyl alcohol (7.6 µL, 1.2 eq.) were dissolved in DMSO-d_6 (0.25 mL) and deoxygenated by bubbling N_2 for 10 min. The polymer mixture was then transferred in a Young's tap NMR tube previously evacuated and refilled with N_2 three times. The catalyst was then added with a degassed syringe and shaken before analysis.

2 Supplementary analysis and experimental data

2.1 Solvent effect on the reaction

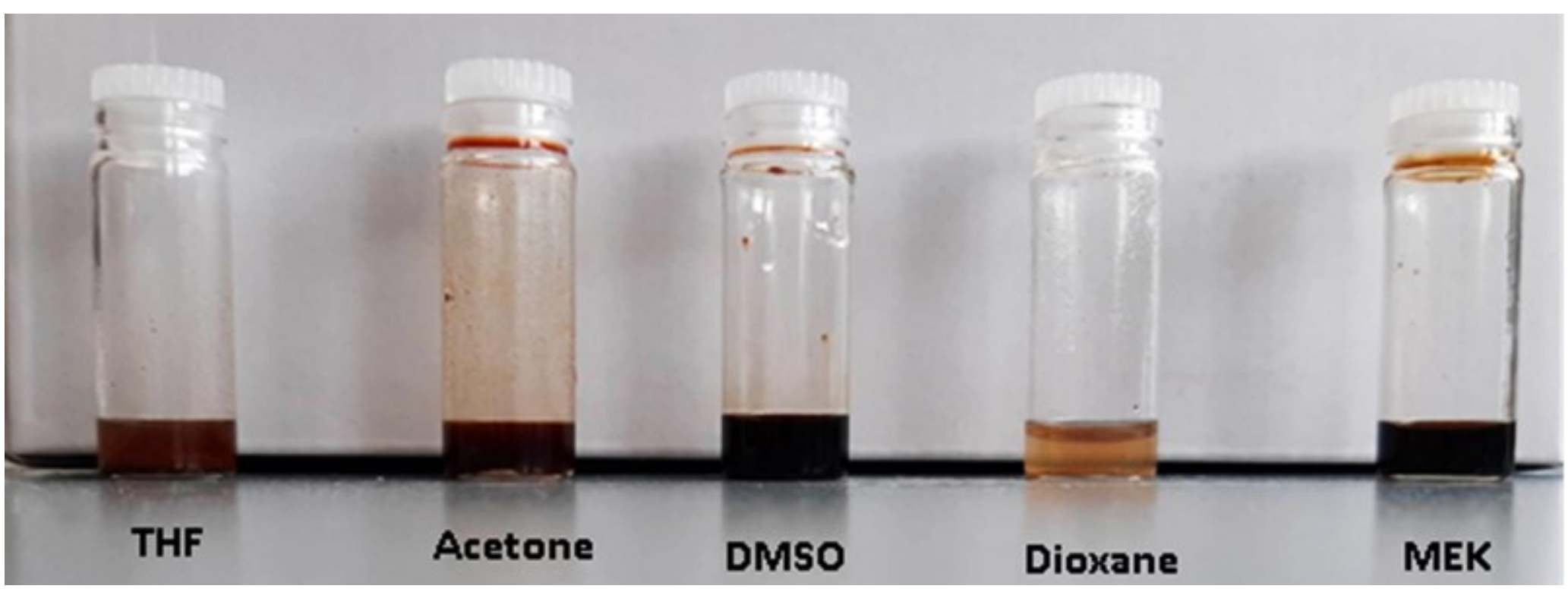

Figure S1. *Cu(I)Br/bpy in 5 different solvents.*

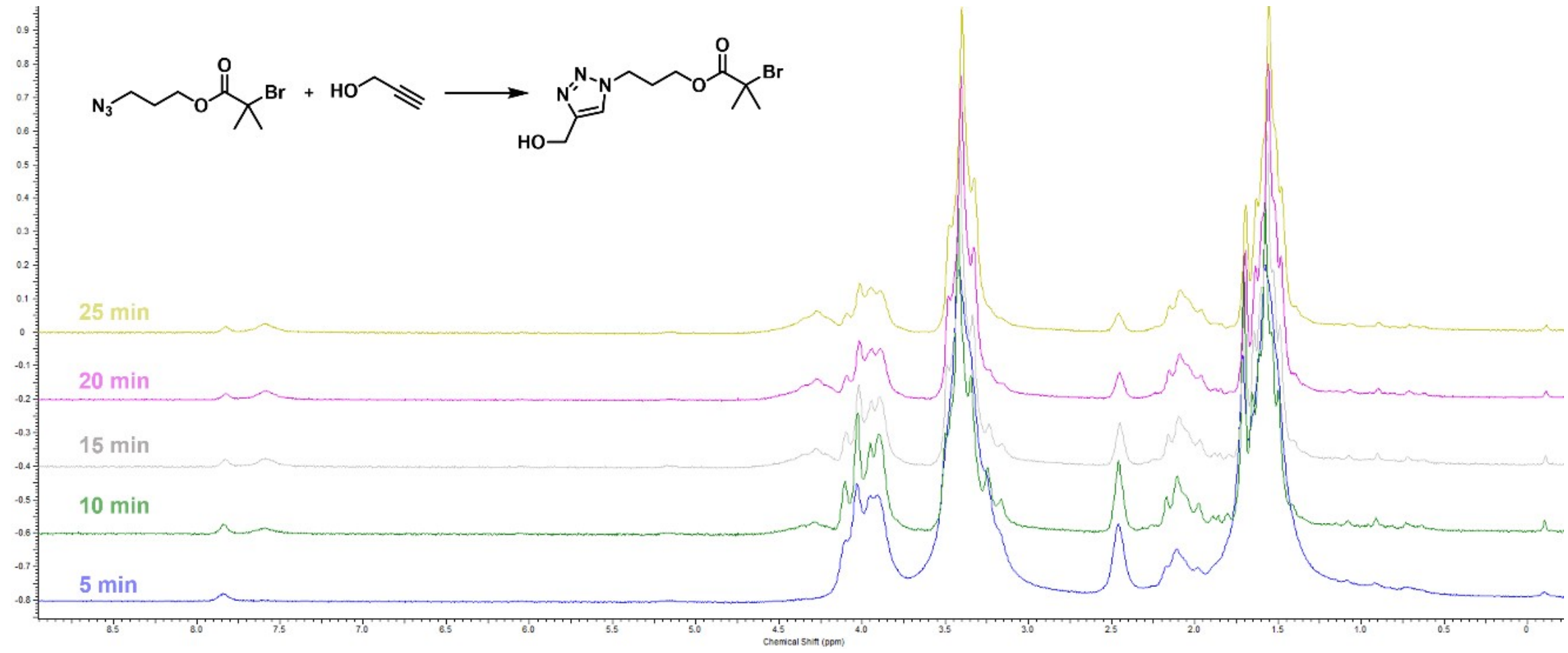

Figure S2. Representative example of the "click" reaction of propargyl alcohol on APBIB in THF monitored with ^{1}H-NMR on the 80 MHz NMR. Lag time due to t_0 = 5 min.

2.2 Effect of the ligand on the reaction rate

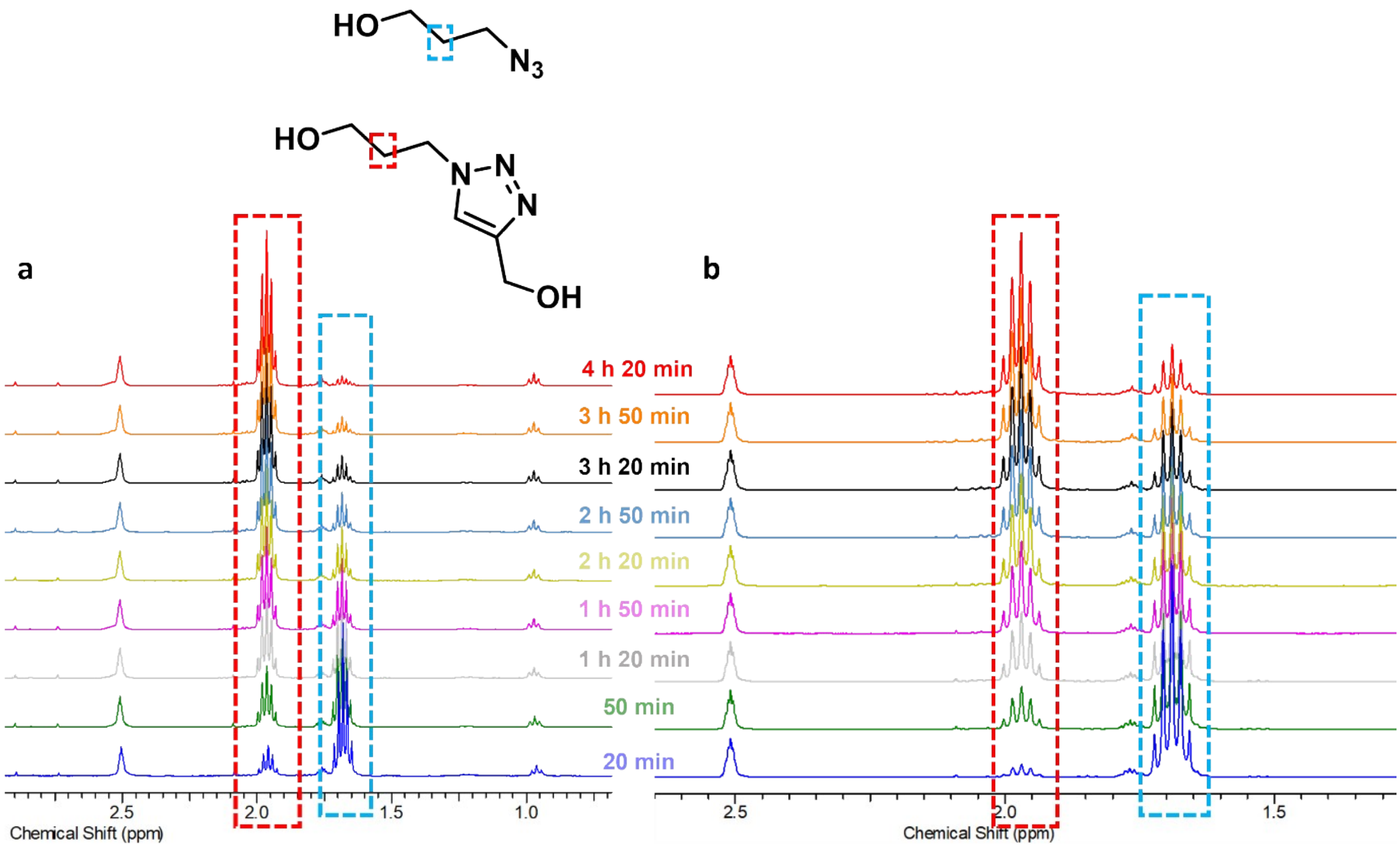

Figure S3. *^{1}H-NMR spectra over a period of 4h with every spectrum taken every 30 min a. with bathophen and b. with bpy ligand in DMSO-d_6.* Lag time due to t_0 = 20 min.

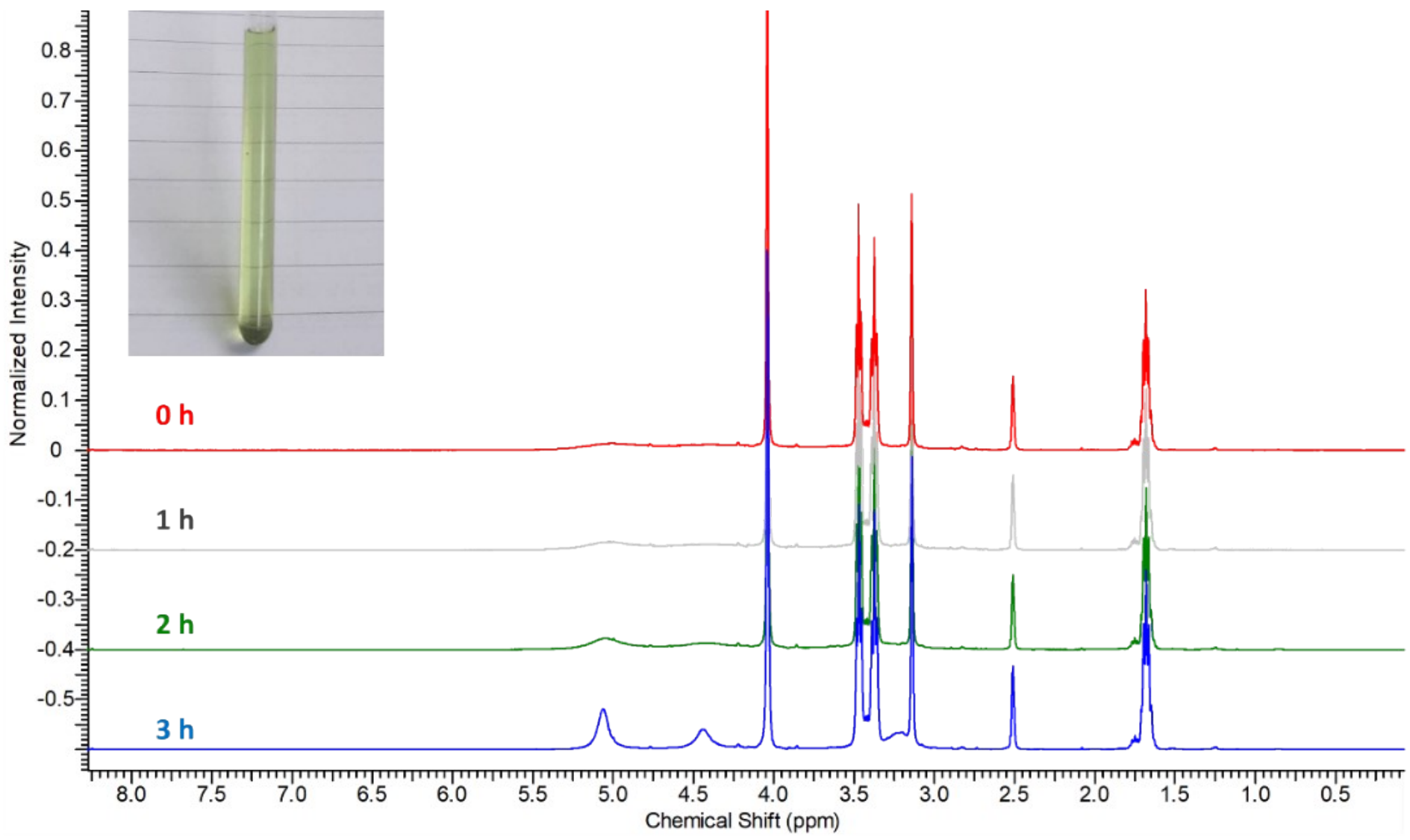

Figure S4. *^{1}H-NMR over time of the reaction in DMSO-d_6 at 50 ℃ with Cu(II)Br_2 and bpy as the catalyst.*

2.3 Effect of the polymer type on the rate of reaction

2.3.1 Azide initiator

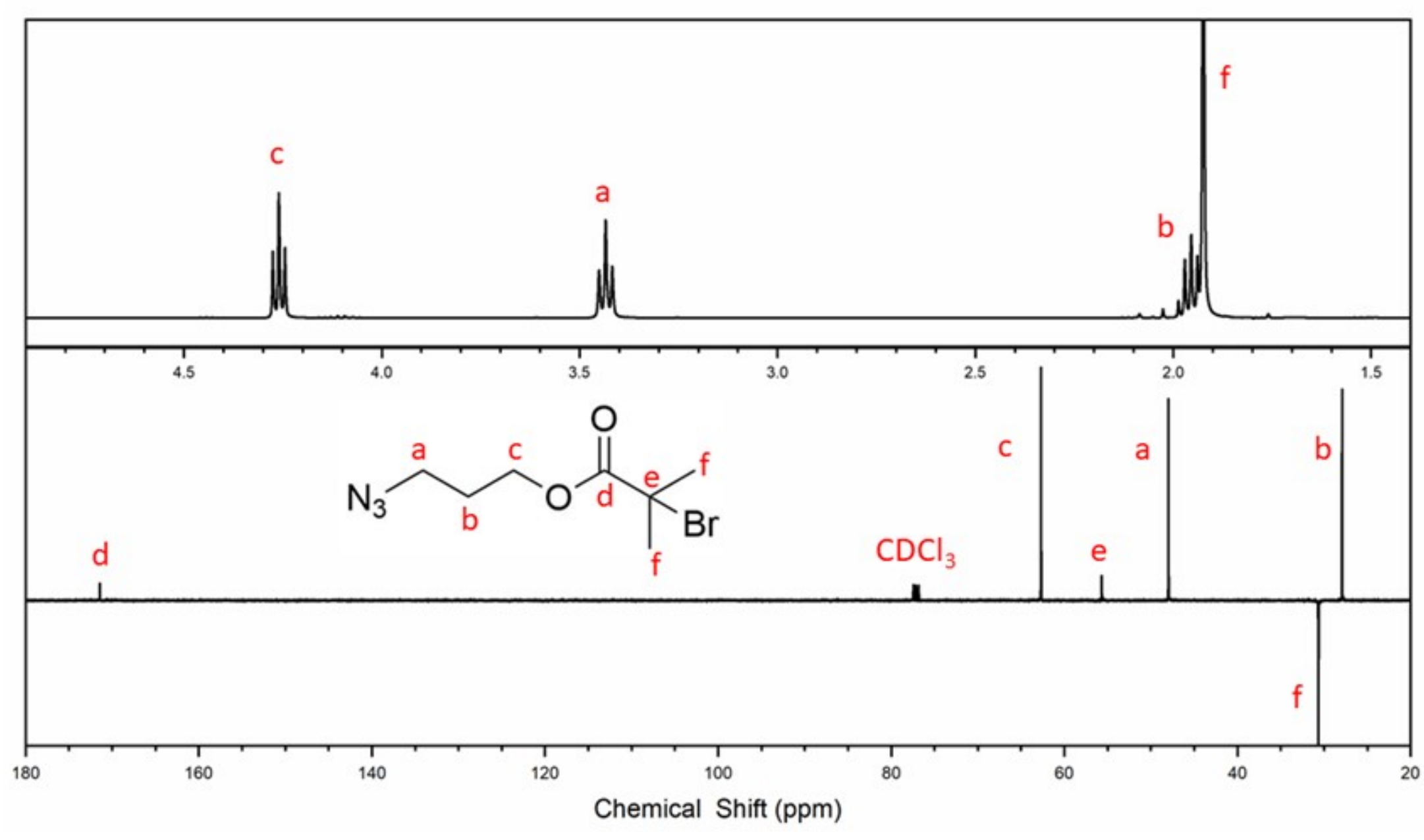

Figure S5. *^{1}H and ^{13}C NMR spectra (400 MHz) of APBIB recorded in $CDCl_3$.*

2.3.2 FTIR of the three polymers

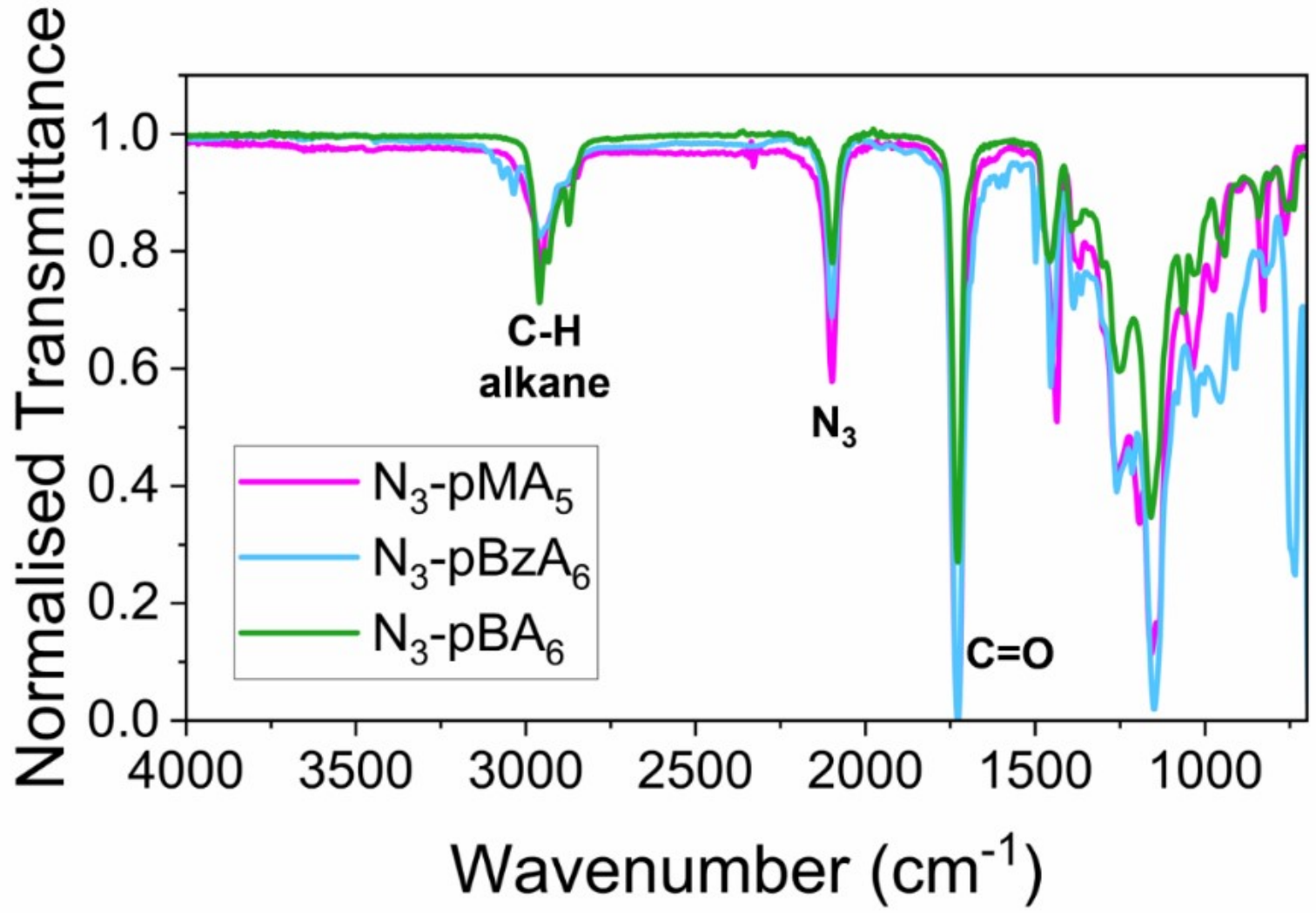

Figure S6. *FTIR spectra of the three azide polymers, N3pMA$_5$, N$_3$pBnA$_6$, N$_3$pBA$_6$.*

2.3.3 DSC analysis of the polymers.

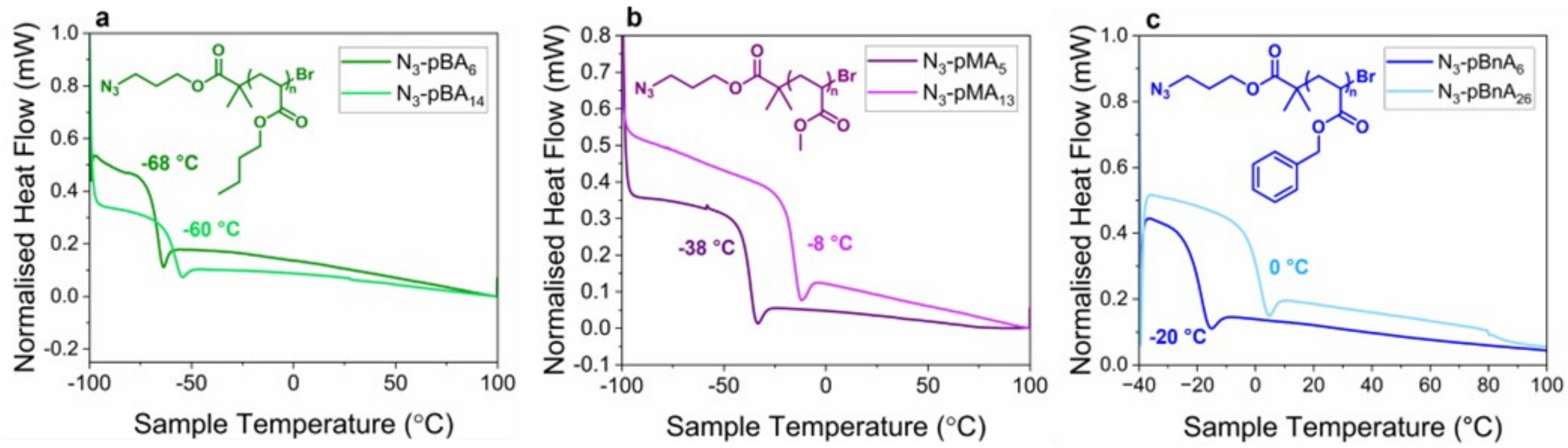

Figure S7. *DSC and glass transitions obtained by the midpoint of the second heating cycle for a) N$_3$pBA$_6$ and N$_3$pBA$_{14}$, b) N$_3$pMA$_5$ and N$_3$pMA$_{13}$ and c) N$_3$pBnA$_6$ and N$_3$pBnA$_{26}$.*

2.5 Results from in batch ^{1}H-NMR monitoring

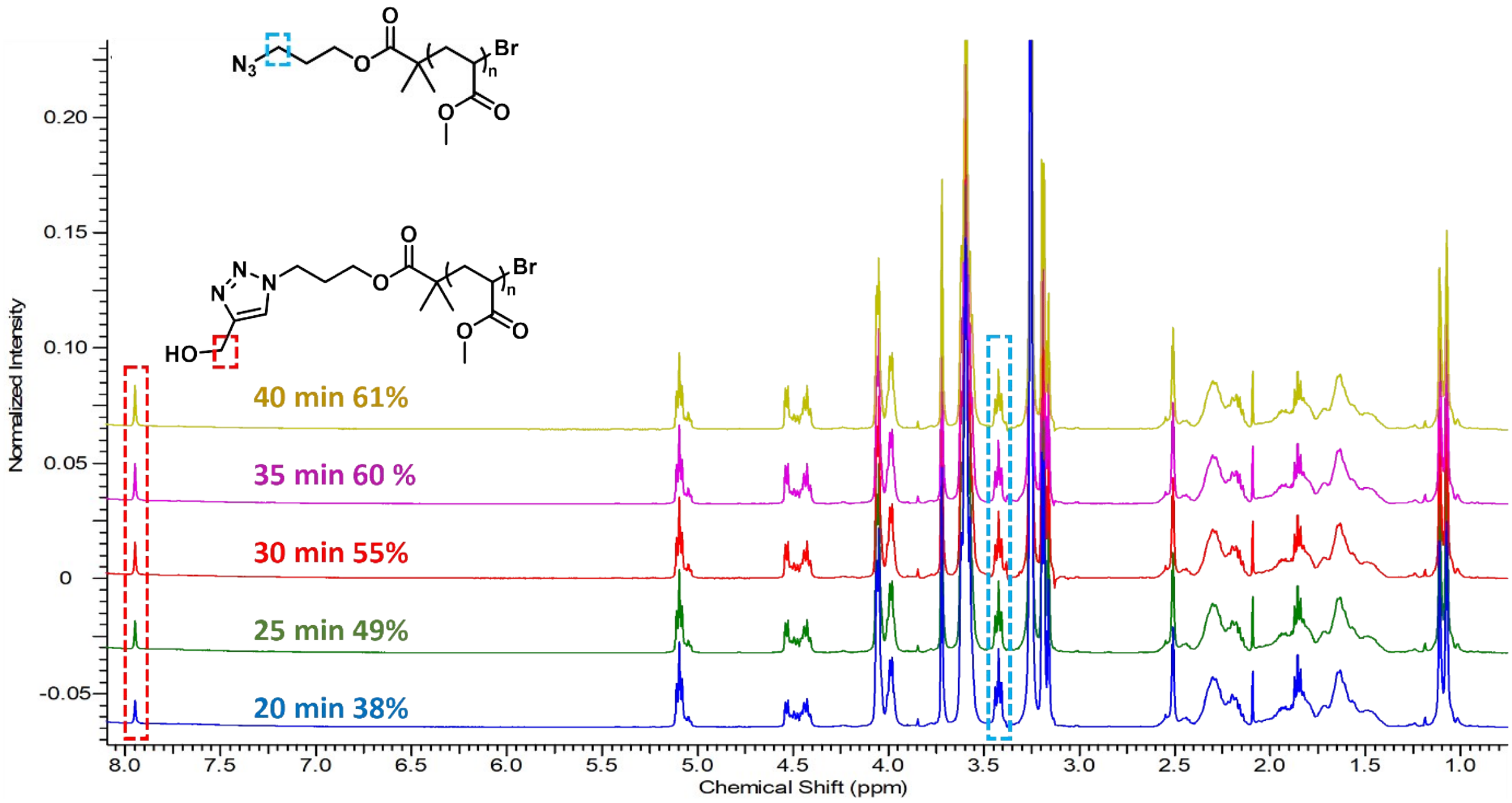

Figure S8. *^{1}H-NMR spectra in DMSO-d_6 at 50 ℃ for the 'click' reaction of N_3pMA_5 (0.06M) with PrOH with reaction conversions at different timepoints. Conversions were found by integrating the proton of the triazole peak and the N_3CH_2 peak of the unreacted polymer. Lag time due to t_0 = 20 min.*

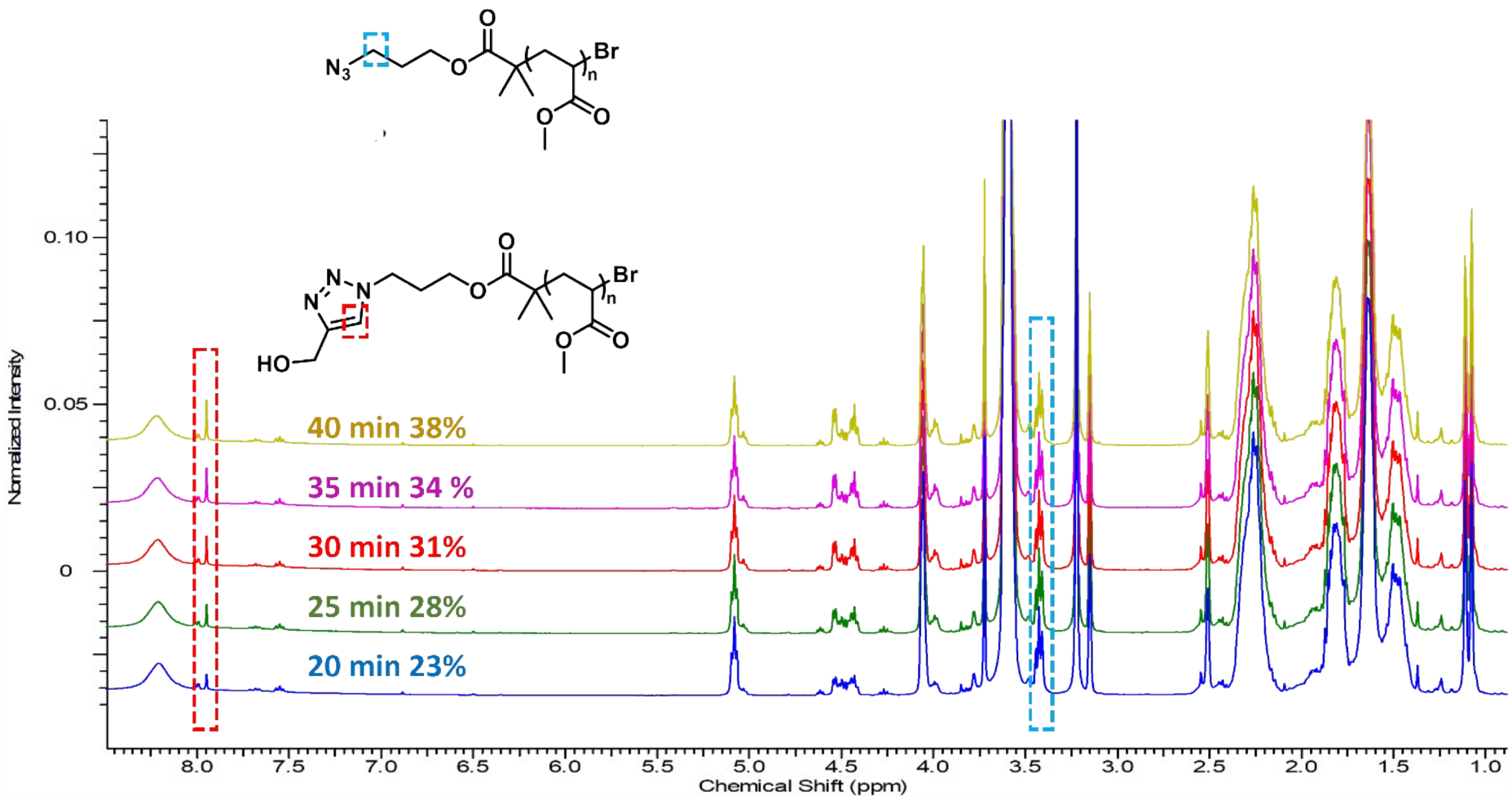

Figure S9. *^{1}H-NMR spectra in DMSO-d_6 at 50 ℃ for the 'click' reaction of N_3pMA_{20} (0.06M) with PrOH with reaction conversions at different timepoints. Conversions were found by integrating the proton of the triazole peak and the N_3CH_2 peak of the unreacted polymer. Lag time due to t_0 = 20 min.*

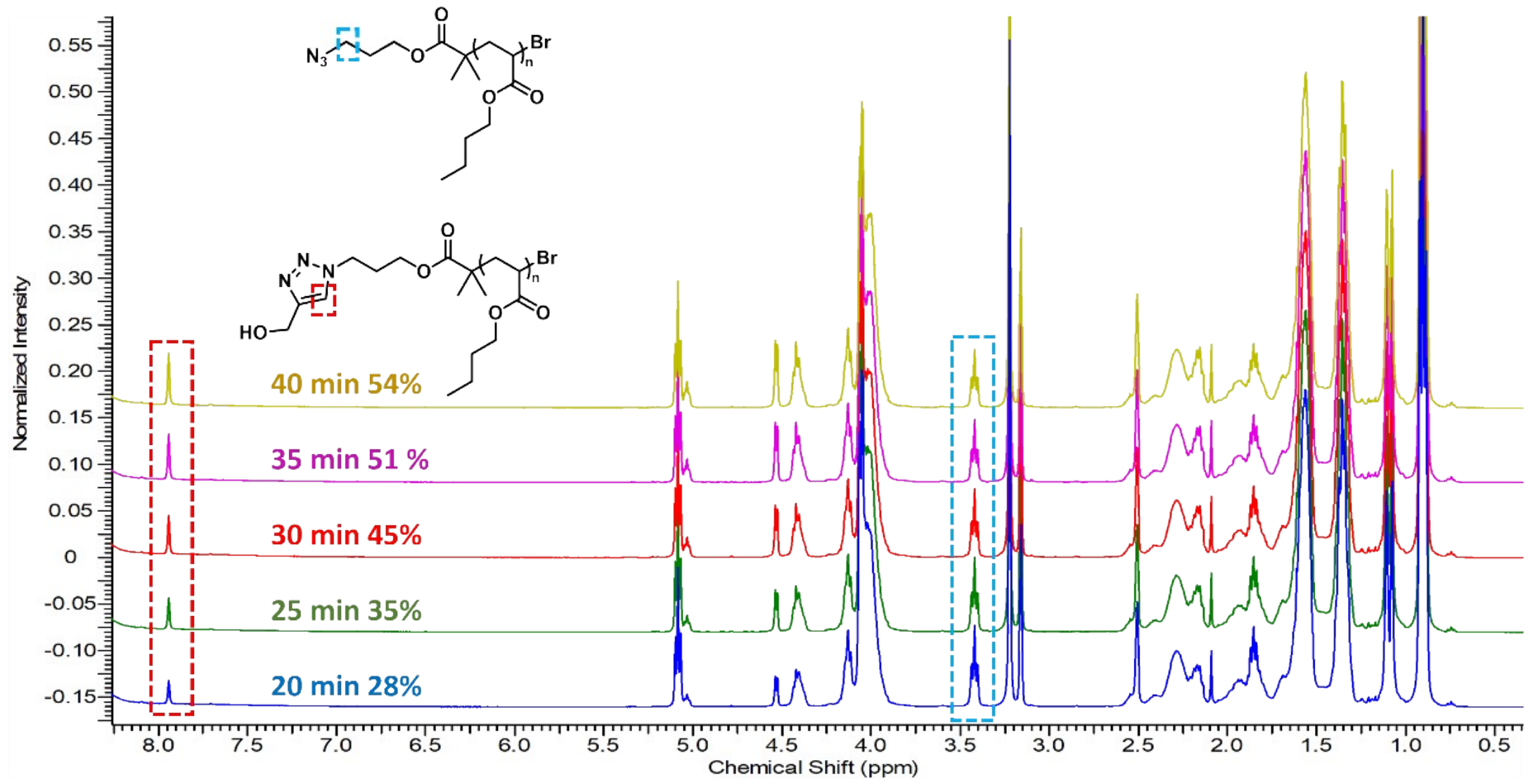

Figure S10. *^{1}H-NMR spectra in DMSO-d_6 at 50 ℃ for the 'click' reaction of N_3pBA_6 (0.06M) with PrOH with reaction conversions at different timepoints. Conversions were found by integrating the proton of the triazole peak and the N_3CH_2 peak of the unreacted polymer. Lag time due to t_0 = 20 min.*

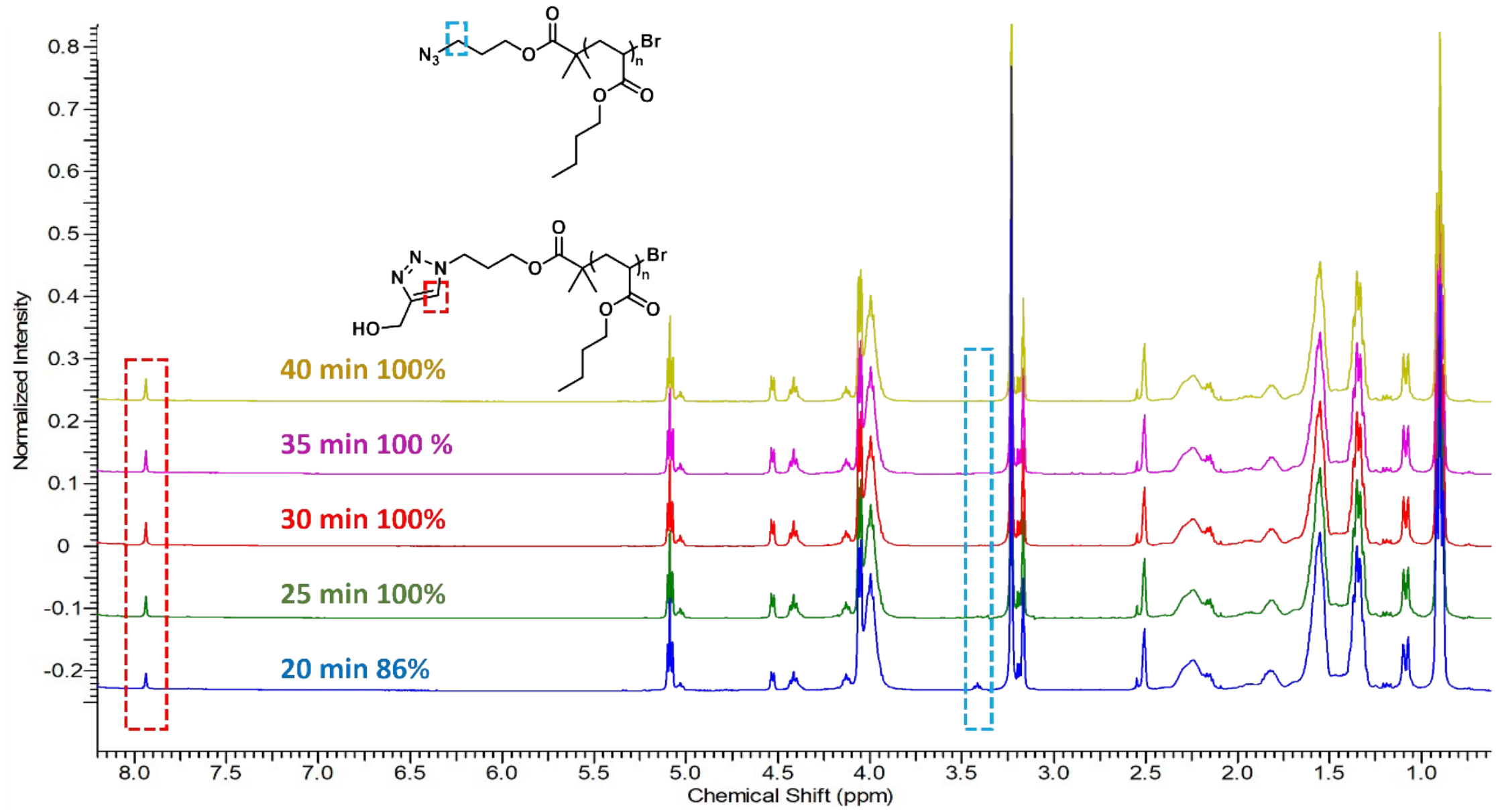

Figure S11. *^{1}H-NMR spectra in DMSO-d_6 at 50 ℃ for the 'click' reaction of N_3pBA_{14} (0.06M) with PrOH with reaction conversions at different timepoints. Conversions were found by integrating the proton of the triazole peak and the N_3CH_2 peak of the unreacted polymer. Lag time due to t_0 = 20 min.*

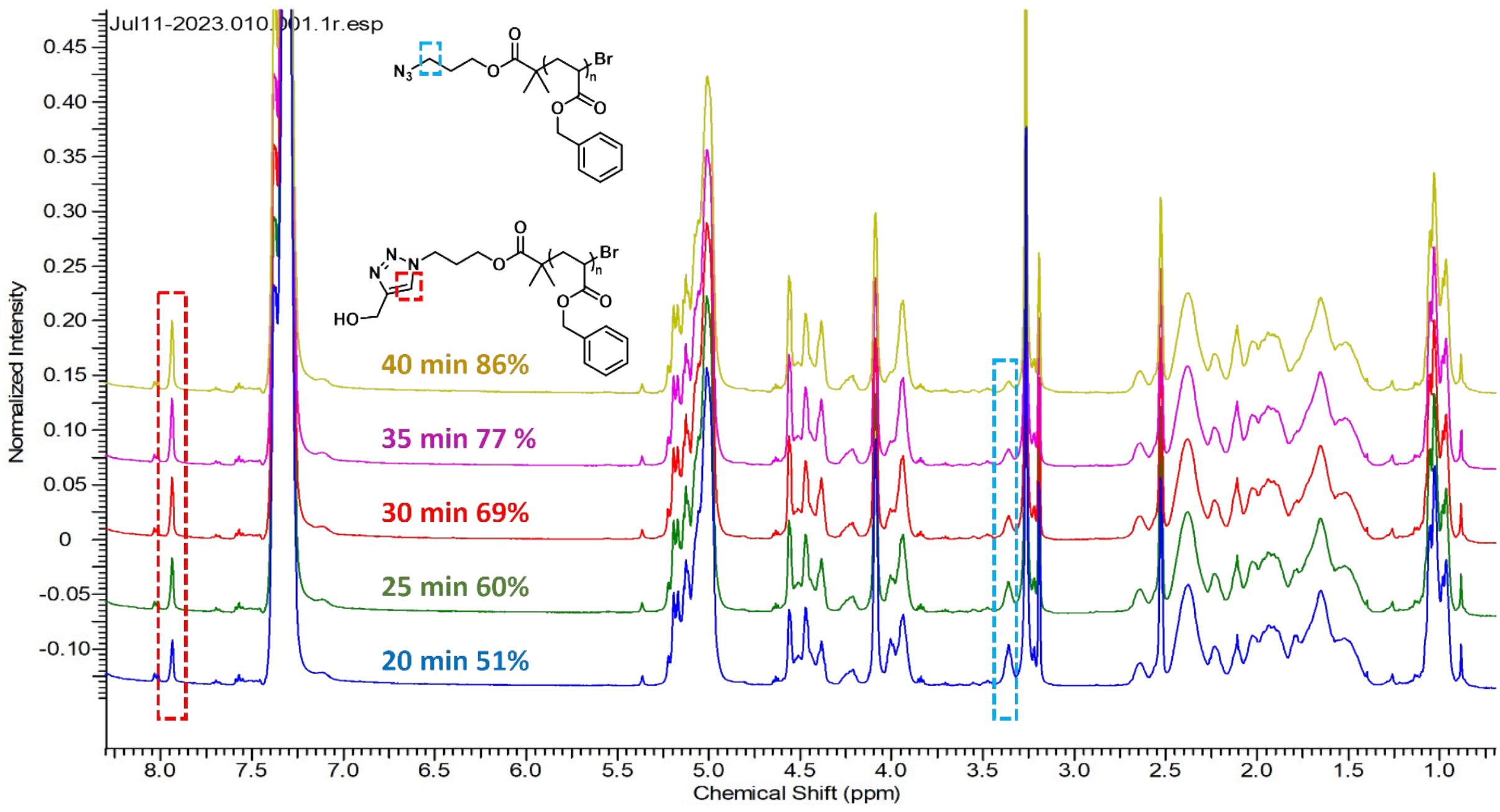

Figure S12. *^{1}H-NMR spectra in DMSO-d_6 at 50 ℃ for the 'click' reaction of N_3pBnA_6 (0.06M) with PrOH with reaction conversions at different timepoints. Conversions were found by integrating the proton of the triazole peak and the N_3CH_2 peak of the unreacted polymer. Lag time due to t_0 = 20 min.*

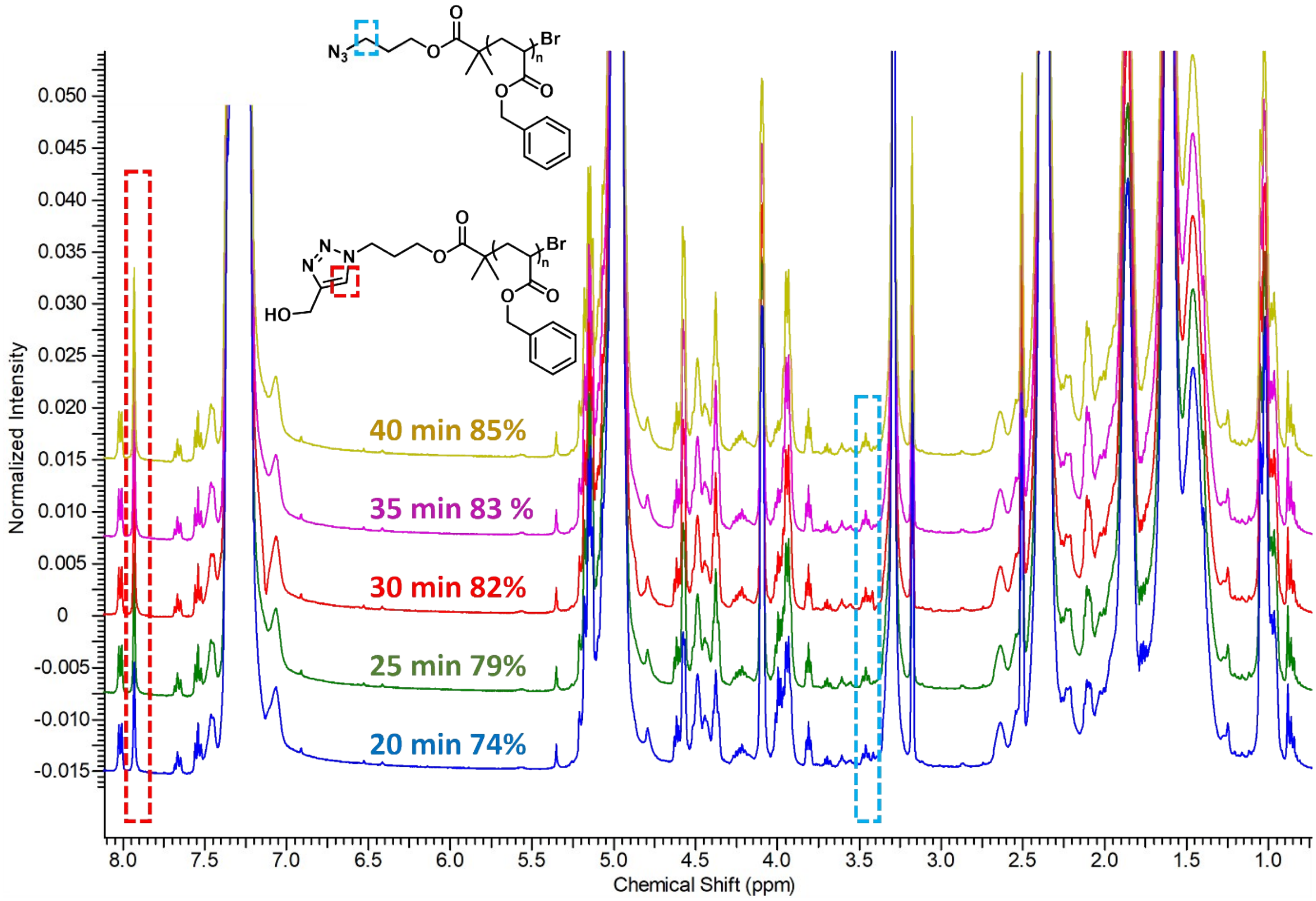

Figure S13. *^{1}H-NMR spectra in DMSO-d_6 at 50 ℃ for the 'click' reaction of N_3pBnA_{26} (0.06M) with PrOH with reaction conversions at different timepoints. Conversions were found by integrating the proton of the triazole peak and the N_3CH_2 peak of the unreacted polymer. Lag time due to t_0 = 20 min.*

2.6 Flow reaction set-up

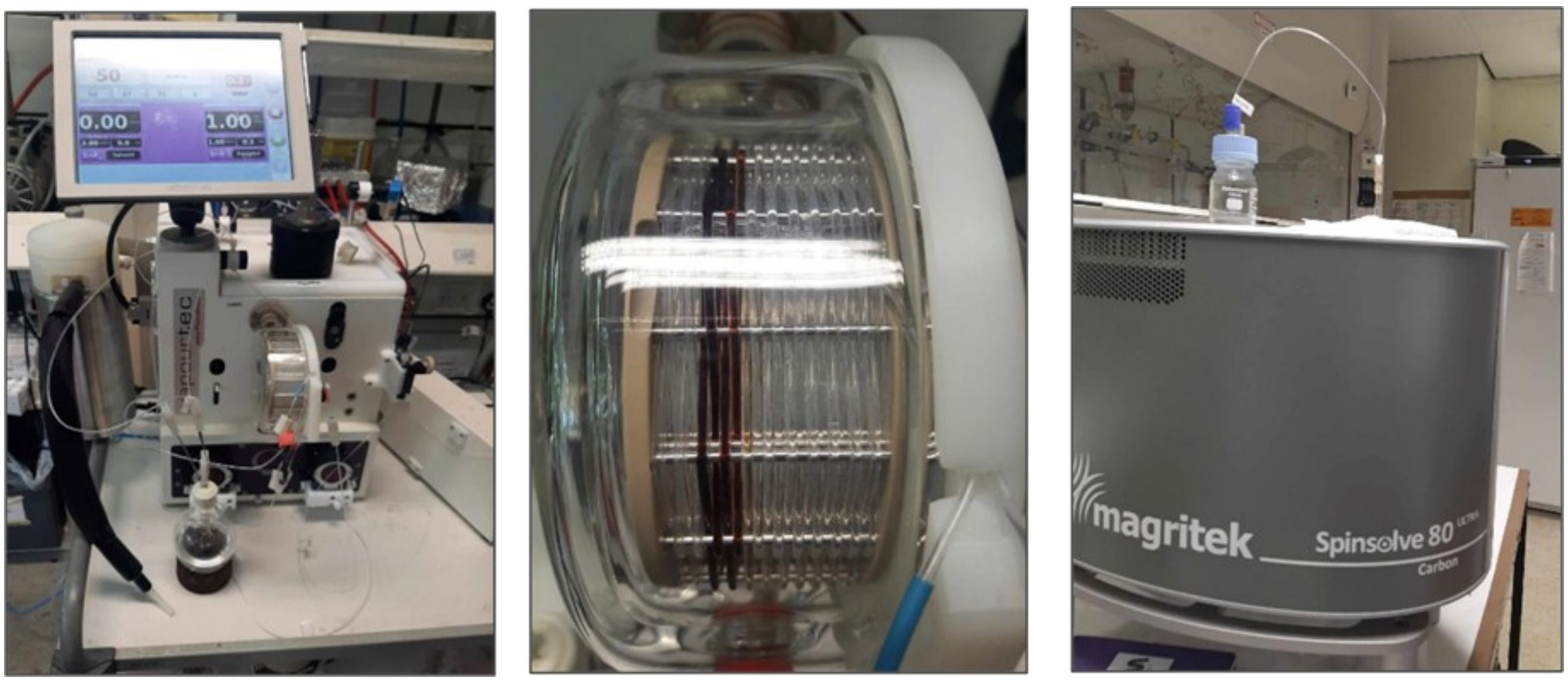

Figure S14. *Flow reaction setup and in-line NMR 80 MHz monitoring. Flow rate 0.25 mL/min and 50 ℃ in the reactor. Total reactor volume was 8 mL with 32 min residence time.*

3 References

1. M. Ciampolini and N. Nardi, *Inorg. Chem.*, 1966, **5**, 41-44.
2. R. N. Keller, H. D. Wrcoff and L. E. Marchi, *Inorg. Synth.*, 1946, 1-4.
3. A. J. Scheel, H. Komber and B. I. Voit, *Macromol. Rapid Commun.*, 2004, **25**, 1175-1180.
4. X. Wu, X. He, L. Zhong, S. Lin, D. Wang, X. Zhu and D. Yan, *J. Mater. Chem.*, 2011, **21**, 13611-13620.
5. H. B. Mereyala and S. R. Gurrala, *Carbohydr. Res.*, 1998, **307**, 351-354.
